# Supplementary material for: AlphaFold Accurately Predicts the Structure of Ribosomally Synthesized and Post-Translationally Modified Peptide Biosynthetic Enzymes
Source: Biomolecules. 2023 Aug 12;13(8):1243. doi: 10.3390/biom13081243 (PMC10452190; doi:10.3390/biom13081243)

Supporting Information for

AlphaFold Accurately Predicts the Structure of RiPP Biosynthetic Enzymes

Figure S1. Comparison of monomer structure predictions of enzymes not involved in RiPP biosynthesis with those that are. Mean TMscores (A) and RMSD (B) are represented by the horizontal lines. Error bars represent standard deviations, and gray circles are values from individual comparisons.

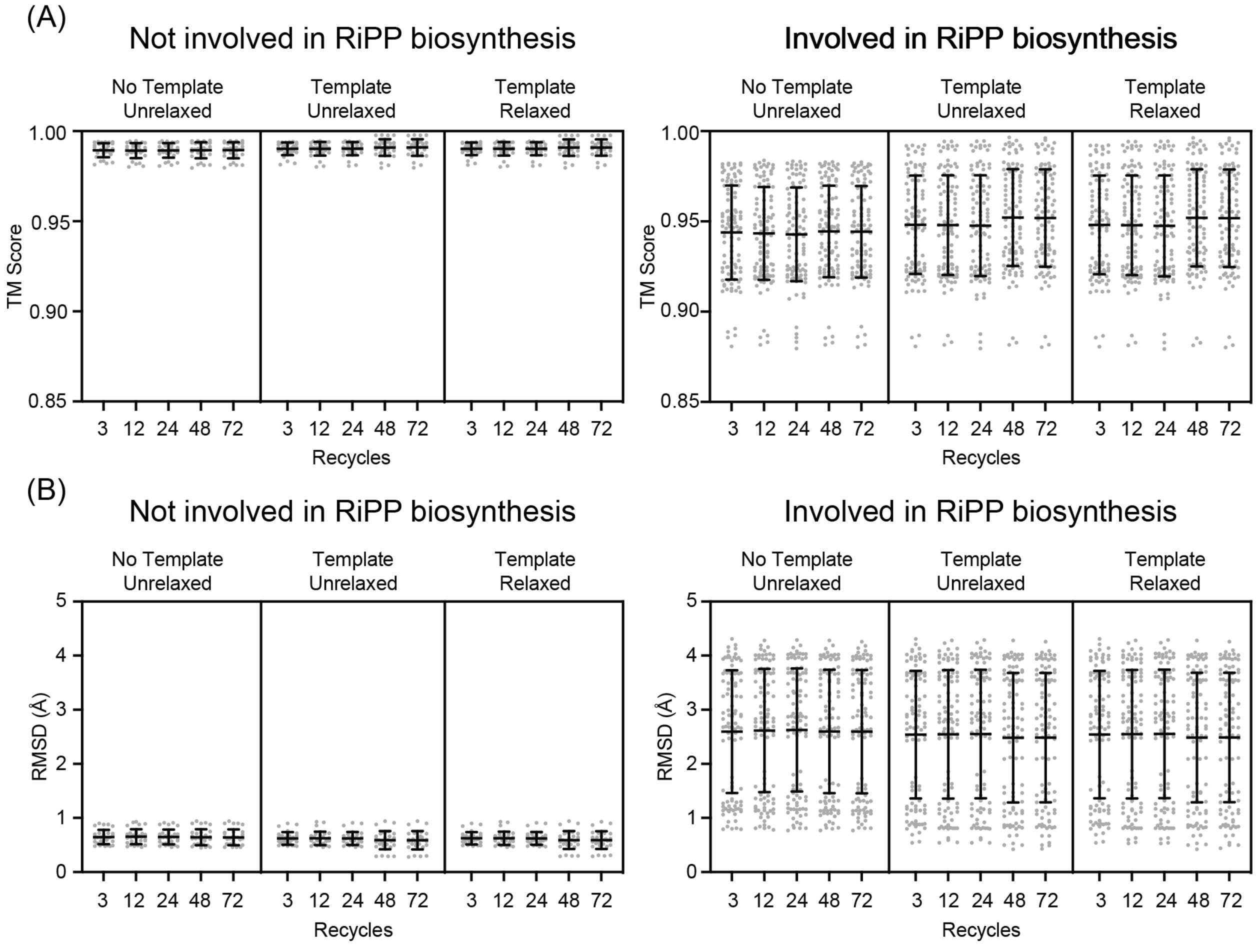

Figure S2. Comparison of monomers in experimental structures for enzymes not involved in RiPP biosynthesis (3VPB and 3VPD) and those that are (5IG8, 5IG9, 7DRM, 7M4S, and 7MGV). Mean TMscores and RMSD are represented by the horizontal lines. Error bars represent standard deviations, and gray circles are values from individual comparisons.

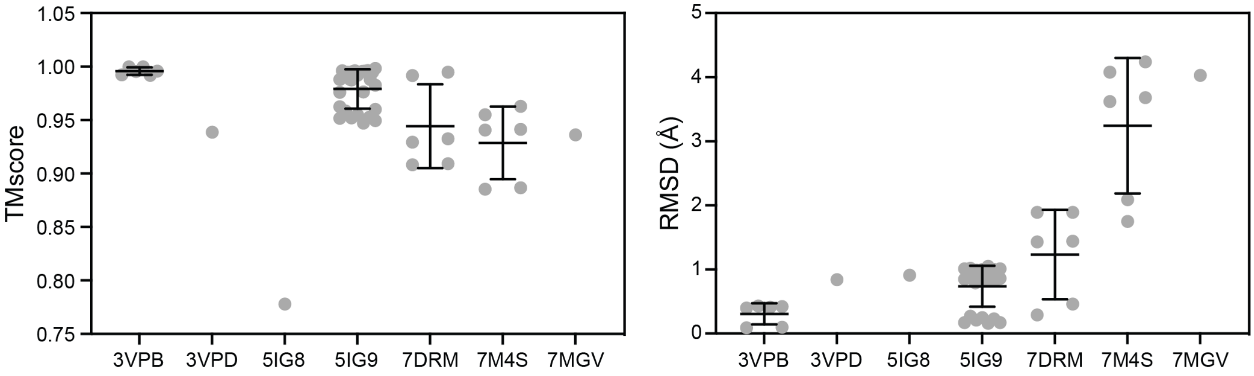

Table S1. Monomer ATP Grasp Ligase RiPP biosynthetic enzyme US-align results without template or AMBER. Enzymes with multiple available reference subunits are denoted with their corresponding chain letters.

PDB ID:	Recycle Number:	Model Number and PDB Reference Chain:	Rank:	TMscore:	RMSD (Å):	Sequence Identity:
3VPB	3	1-A	3	0.9918	0.57	1.000
3VPB	3	2-A	5	0.9902	0.62	1.000
3VPB	3	3-A	1	0.9918	0.57	1.000
3VPB	3	4-A	4	0.9885	0.67	1.000
3VPB	3	5-A	2	0.9896	0.64	1.000
3VPB	3	1-B	3	0.9896	0.64	1.000
3VPB	3	2-B	5	0.9897	0.64	1.000
3VPB	3	3-B	1	0.9911	0.59	1.000
3VPB	3	4-B	4	0.9879	0.70	1.000
3VPB	3	5-B	2	0.9889	0.67	1.000
3VPB	3	1-C	3	0.9929	0.53	1.000
3VPB	3	2-C	5	0.9938	0.49	1.000
3VPB	3	3-C	1	0.9939	0.49	1.000
3VPB	3	4-C	4	0.9939	0.49	1.000
3VPB	3	5-C	2	0.9917	0.57	1.000
3VPB	3	1-D	3	0.9931	0.52	1.000
3VPB	3	2-D	5	0.9940	0.48	1.000
3VPB	3	3-D	1	0.9931	0.52	1.000
3VPB	3	4-D	4	0.9940	0.48	1.000
3VPB	3	5-D	2	0.9922	0.56	1.000
3VPD	3	1-A	5	0.9826	0.88	1.000
3VPD	3	2-A	4	0.9834	0.86	1.000
3VPD	3	3-A	1	0.9822	0.88	1.000
3VPD	3	4-A	2	0.9833	0.85	1.000
3VPD	3	5-A	3	0.9814	0.90	1.000
3VPD	3	1-B	5	0.9871	0.72	1.000
3VPD	3	2-B	4	0.9886	0.69	1.000
3VPD	3	3-B	1	0.9882	0.71	1.000
3VPD	3	4-B	2	0.9876	0.72	1.000
3VPD	3	5-B	3	0.9857	0.76	1.000
5IG8	3	1-A	5	0.9823	0.79	1.000

PDB ID:	Recycle Number:	Model Number and PDB Reference Chain:	Rank:	TMscore:	RMSD (Å):	Sequence Identity:
5IG8	3	2-A	4	0.9821	0.79	1.000
5IG8	3	3-A	1	0.9818	0.80	1.000
5IG8	3	4-A	3	0.9813	0.81	1.000
5IG8	3	5-A	2	0.9801	0.84	1.000
5IG8	3	1-B	5	0.9823	0.79	1.000
5IG8	3	2-B	4	0.9821	0.79	1.000
5IG8	3	3-B	1	0.9818	0.80	1.000
5IG8	3	4-B	3	0.9813	0.81	1.000
5IG8	3	5-B	2	0.9801	0.84	1.000
5IG9	3	1-A	5	0.9403	3.97	0.981
5IG9	3	2-A	3	0.9471	3.91	0.981
5IG9	3	3-A	1	0.9450	3.94	0.981
5IG9	3	4-A	4	0.9411	3.96	0.981
5IG9	3	5-A	2	0.9465	3.94	0.981
5IG9	3	1-B	5	0.9659	1.35	0.980
5IG9	3	2-B	3	0.9722	1.22	0.980
5IG9	3	3-B	1	0.9726	1.22	0.980
5IG9	3	4-B	4	0.9678	1.32	0.980
5IG9	3	5-B	2	0.9749	1.17	0.980
5IG9	3	1-C	5	0.9493	3.29	0.984
5IG9	3	2-C	3	0.9556	3.24	0.984
5IG9	3	3-C	1	0.9552	3.25	0.984
5IG9	3	4-C	4	0.9528	3.25	0.984
5IG9	3	5-C	2	0.9563	3.25	0.984
5IG9	3	1-D	5	0.9699	1.30	0.984
5IG9	3	2-D	3	0.9763	1.17	0.984
5IG9	3	3-D	1	0.9771	1.15	0.984
5IG9	3	4-D	4	0.9728	1.23	0.984
5IG9	3	5-D	2	0.9780	1.14	0.984
5IG9	3	1-E	5	0.9450	3.72	0.984
5IG9	3	2-E	3	0.9515	3.67	0.984
5IG9	3	3-E	1	0.9511	3.68	0.984
5IG9	3	4-E	4	0.9487	3.69	0.984
5IG9	3	5-E	2	0.9521	3.68	0.984
5IG9	3	1-F	5	0.9722	1.29	0.983
5IG9	3	2-F	3	0.9783	1.16	0.983
5IG9	3	3-F	1	0.9791	1.16	0.983
5IG9	3	4-F	4	0.9760	1.21	0.983
5IG9	3	5-F	2	0.9800	1.14	0.983
5IG9	3	1-G	5	0.9472	3.71	0.984
5IG9	3	2-G	3	0.9528	3.67	0.984
5IG9	3	3-G	1	0.9530	3.67	0.984
5IG9	3	4-G	4	0.9507	3.68	0.984
5IG9	3	5-G	2	0.9543	3.67	0.984
5IG9	3	1-H	5	0.9634	1.30	0.981
5IG9	3	2-H	3	0.9698	1.14	0.981
5IG9	3	3-H	1	0.9710	1.12	0.981
5IG9	3	4-H	4	0.9654	1.27	0.981
5IG9	3	5-H	2	0.9729	1.07	0.981
7DRM	3	1-A	5	0.9384	2.57	1.000
7DRM	3	2-A	3	0.9438	2.47	1.000
7DRM	3	3-A	1	0.9474	2.44	1.000
7DRM	3	4-A	2	0.9311	2.68	1.000
7DRM	3	5-A	4	0.9242	2.68	1.000
7DRM	3	1-B	5	0.9551	1.54	1.000
7DRM	3	2-B	3	0.9542	1.55	1.000
7DRM	3	3-B	1	0.9559	1.51	1.000
7DRM	3	4-B	2	0.9464	1.75	1.000
7DRM	3	5-B	4	0.9501	1.65	1.000
7DRM	3	1-C	5	0.9364	2.57	1.000
7DRM	3	2-C	3	0.9418	2.47	1.000
7DRM	3	3-C	1	0.9461	2.43	1.000
7DRM	3	4-C	2	0.9296	2.66	1.000
7DRM	3	5-C	4	0.9220	2.68	1.000

PDB ID:	Recycle Number:	Model Number and PDB Reference Chain:	Rank:	TMscore:	RMSD (Å):	Sequence Identity:
7DRM	3	1-D	5	0.9243	2.93	0.994
7DRM	3	2-D	3	0.9248	2.88	0.994
7DRM	3	3-D	1	0.9261	2.90	0.994
7DRM	3	4-D	2	0.9150	3.07	0.994
7DRM	3	5-D	4	0.9173	2.99	0.994
7M4S	3	1-A	5	0.9124	2.91	1.000
7M4S	3	2-A	2	0.9135	2.88	1.000
7M4S	3	3-A	1	0.9177	2.83	1.000
7M4S	3	4-A	3	0.9115	2.94	1.000
7M4S	3	5-A	4	0.9116	2.98	1.000
7M4S	3	1-B	5	0.9129	2.69	1.000
7M4S	3	2-B	2	0.9143	2.68	1.000
7M4S	3	3-B	1	0.9166	2.64	1.000
7M4S	3	4-B	3	0.9113	2.75	1.000
7M4S	3	5-B	4	0.9108	2.75	1.000
7M4S	3	1-C	5	0.9192	3.49	1.000
7M4S	3	2-C	2	0.9209	3.49	1.000
7M4S	3	3-C	1	0.9220	3.44	1.000
7M4S	3	4-C	3	0.9173	3.54	1.000
7M4S	3	5-C	4	0.9154	3.58	1.000
7M4S	3	1-D	5	0.9283	3.97	1.000
7M4S	3	2-D	2	0.9314	3.97	1.000
7M4S	3	3-D	1	0.9335	3.91	1.000
7M4S	3	4-D	3	0.9274	4.00	1.000
7M4S	3	5-D	4	0.9272	4.03	1.000
7MGV	3	1-A	3	0.9214	4.16	1.000
7MGV	3	2-A	5	0.9180	4.20	1.000
7MGV	3	3-A	1	0.9247	4.08	1.000
7MGV	3	4-A	4	0.9229	4.31	1.000
7MGV	3	5-A	2	0.9245	4.13	1.000
7MGV	3	1-B	3	0.9768	1.06	1.000
7MGV	3	2-B	5	0.9741	1.12	1.000
7MGV	3	3-B	1	0.9813	0.94	1.000
7MGV	3	4-B	4	0.9619	1.58	1.000
7MGV	3	5-B	2	0.9818	0.93	1.000
3VPB	12	1-A	4	0.9898	0.64	1.000
3VPB	12	2-A	5	0.9895	0.65	1.000
3VPB	12	3-A	1	0.9915	0.58	1.000
3VPB	12	4-A	3	0.9901	0.63	1.000
3VPB	12	5-A	2	0.9886	0.68	1.000
3VPB	12	1-B	4	0.9894	0.65	1.000
3VPB	12	2-B	5	0.9889	0.67	1.000
3VPB	12	3-B	1	0.9909	0.60	1.000
3VPB	12	4-B	3	0.9895	0.65	1.000
3VPB	12	5-B	2	0.9880	0.70	1.000
3VPB	12	1-C	4	0.9926	0.54	1.000
3VPB	12	2-C	5	0.9937	0.49	1.000
3VPB	12	3-C	1	0.9935	0.50	1.000
3VPB	12	4-C	3	0.9944	0.47	1.000
3VPB	12	5-C	2	0.9916	0.58	1.000
3VPB	12	1-D	4	0.9929	0.53	1.000
3VPB	12	2-D	5	0.9940	0.48	1.000
3VPB	12	3-D	1	0.9939	0.49	1.000
3VPB	12	4-D	3	0.9946	0.46	1.000
3VPB	12	5-D	2	0.9918	0.57	1.000
3VPD	12	1-A	5	0.9818	0.89	1.000
3VPD	12	2-A	4	0.9822	0.88	1.000
3VPD	12	3-A	1	0.9802	0.93	1.000
3VPD	12	4-A	2	0.9829	0.86	1.000
3VPD	12	5-A	3	0.9806	0.92	1.000
3VPD	12	1-B	5	0.9872	0.72	1.000
3VPD	12	2-B	4	0.9884	0.69	1.000
3VPD	12	3-B	1	0.9878	0.72	1.000
3VPD	12	4-B	2	0.9881	0.71	1.000

PDB ID:	Recycle Number:	Model Number and PDB Reference Chain:	Rank:	TMscore:	RMSD (Å):	Sequence Identity:
3VPD	12	5-B	3	0.9864	0.74	1.000
5IG8	12	1-A	5	0.8858	3.76	1.000
5IG8	12	2-A	4	0.8895	3.41	1.000
5IG8	12	3-A	1	0.8831	3.91	1.000
5IG8	12	4-A	3	0.8816	3.94	1.000
5IG8	12	5-A	2	0.8868	3.72	1.000
5IG8	12	1-B	5	0.9814	0.81	1.000
5IG8	12	2-B	4	0.9825	0.78	1.000
5IG8	12	3-B	1	0.9813	0.81	1.000
5IG8	12	4-B	3	0.9798	0.84	1.000
5IG8	12	5-B	2	0.9804	0.83	1.000
5IG9	12	1-A	5	0.9431	3.96	0.981
5IG9	12	2-A	4	0.9473	3.93	0.981
5IG9	12	3-A	1	0.9431	3.96	0.981
5IG9	12	4-A	3	0.9369	4.03	0.981
5IG9	12	5-A	2	0.9481	3.94	0.981
5IG9	12	1-B	5	0.9617	1.43	0.980
5IG9	12	2-B	4	0.9727	1.21	0.980
5IG9	12	3-B	1	0.9709	1.26	0.980
5IG9	12	4-B	3	0.9639	1.41	0.980
5IG9	12	5-B	2	0.9764	1.13	0.980
5IG9	12	1-C	5	0.9464	3.32	0.984
5IG9	12	2-C	4	0.9567	3.24	0.984
5IG9	12	3-C	1	0.9540	3.26	0.984
5IG9	12	4-C	3	0.9501	3.31	0.984
5IG9	12	5-C	2	0.9577	3.25	0.984
5IG9	12	1-D	5	0.9666	1.36	0.984
5IG9	12	2-D	4	0.9774	1.14	0.984
5IG9	12	3-D	1	0.9756	1.19	0.984
5IG9	12	4-D	3	0.9691	1.31	0.984
5IG9	12	5-D	2	0.9801	1.08	0.984
5IG9	12	1-E	5	0.9422	3.76	0.984
5IG9	12	2-E	4	0.9526	3.67	0.984
5IG9	12	3-E	1	0.9499	3.69	0.984
5IG9	12	4-E	3	0.9463	3.74	0.984
5IG9	12	5-E	2	0.9537	3.68	0.984
5IG9	12	1-F	5	0.9694	1.35	0.983
5IG9	12	2-F	4	0.9795	1.14	0.983
5IG9	12	3-F	1	0.9780	1.18	0.983
5IG9	12	4-F	3	0.9731	1.27	0.983
5IG9	12	5-F	2	0.9820	1.09	0.983
5IG9	12	1-G	5	0.9445	3.75	0.984
5IG9	12	2-G	4	0.9539	3.67	0.984
5IG9	12	3-G	1	0.9519	3.69	0.984
5IG9	12	4-G	3	0.9481	3.73	0.984
5IG9	12	5-G	2	0.9557	3.68	0.984
5IG9	12	1-H	5	0.9599	1.37	0.981
5IG9	12	2-H	4	0.9704	1.13	0.981
5IG9	12	3-H	1	0.9693	1.16	0.981
5IG9	12	4-H	3	0.9625	1.34	0.981
5IG9	12	5-H	2	0.9748	1.02	0.981
7DRM	12	1-A	4	0.9287	2.69	1.000
7DRM	12	2-A	3	0.9404	2.53	1.000
7DRM	12	3-A	1	0.9445	2.49	1.000
7DRM	12	4-A	2	0.9264	2.77	1.000
7DRM	12	5-A	5	0.9166	2.77	1.000
7DRM	12	1-B	4	0.9524	1.59	1.000
7DRM	12	2-B	3	0.9520	1.58	1.000
7DRM	12	3-B	1	0.9567	1.51	1.000
7DRM	12	4-B	2	0.9422	1.86	1.000
7DRM	12	5-B	5	0.9454	1.73	1.000
7DRM	12	1-C	4	0.9266	2.69	1.000
7DRM	12	2-C	3	0.9383	2.53	1.000
7DRM	12	3-C	1	0.9431	2.48	1.000

PDB ID:	Recycle Number:	Model Number and PDB Reference Chain:	Rank:	TMscore:	RMSD (Å):	Sequence Identity:
7DRM	12	4-C	2	0.9249	2.73	1.000
7DRM	12	5-C	5	0.9144	2.77	1.000
7DRM	12	1-D	4	0.9198	3.00	0.994
7DRM	12	2-D	3	0.9216	2.94	0.994
7DRM	12	3-D	1	0.9260	2.92	0.994
7DRM	12	4-D	2	0.9104	3.15	0.994
7DRM	12	5-D	5	0.9122	3.06	0.994
7M4S	12	1-A	5	0.9182	2.85	1.000
7M4S	12	2-A	2	0.9159	2.87	1.000
7M4S	12	3-A	1	0.9187	2.84	1.000
7M4S	12	4-A	3	0.9169	2.83	1.000
7M4S	12	5-A	4	0.9189	2.78	1.000
7M4S	12	1-B	5	0.9199	2.60	1.000
7M4S	12	2-B	2	0.9176	2.65	1.000
7M4S	12	3-B	1	0.9196	2.59	1.000
7M4S	12	4-B	3	0.9162	2.65	1.000
7M4S	12	5-B	4	0.9198	2.55	1.000
7M4S	12	1-C	5	0.9231	3.52	1.000
7M4S	12	2-C	2	0.9227	3.52	1.000
7M4S	12	3-C	1	0.9227	3.46	1.000
7M4S	12	4-C	3	0.9205	3.53	1.000
7M4S	12	5-C	4	0.9198	3.56	1.000
7M4S	12	1-D	5	0.9326	4.01	1.000
7M4S	12	2-D	2	0.9323	4.01	1.000
7M4S	12	3-D	1	0.9333	3.94	1.000
7M4S	12	4-D	3	0.9304	4.01	1.000
7M4S	12	5-D	4	0.9331	4.01	1.000
7MGV	12	1-A	4	0.9230	4.16	1.000
7MGV	12	2-A	5	0.9196	4.19	1.000
7MGV	12	3-A	1	0.9247	4.09	1.000
7MGV	12	4-A	3	0.9245	4.28	1.000
7MGV	12	5-A	2	0.9264	4.12	1.000
7MGV	12	1-B	4	0.9788	1.01	1.000
7MGV	12	2-B	5	0.9754	1.09	1.000
7MGV	12	3-B	1	0.9814	0.94	1.000
7MGV	12	4-B	3	0.9618	1.63	1.000
7MGV	12	5-B	2	0.9838	0.87	1.000
3VPB	24	1-A	4	0.9897	0.64	1.000
3VPB	24	2-A	5	0.9897	0.64	1.000
3VPB	24	3-A	1	0.9914	0.58	1.000
3VPB	24	4-A	3	0.9903	0.62	1.000
3VPB	24	5-A	2	0.9886	0.67	1.000
3VPB	24	1-B	4	0.9893	0.65	1.000
3VPB	24	2-B	5	0.9892	0.66	1.000
3VPB	24	3-B	1	0.9909	0.60	1.000
3VPB	24	4-B	3	0.9898	0.64	1.000
3VPB	24	5-B	2	0.9881	0.70	1.000
3VPB	24	1-C	4	0.9928	0.53	1.000
3VPB	24	2-C	5	0.9937	0.50	1.000
3VPB	24	3-C	1	0.9936	0.50	1.000
3VPB	24	4-C	3	0.9944	0.47	1.000
3VPB	24	5-C	2	0.9918	0.57	1.000
3VPB	24	1-D	4	0.9931	0.52	1.000
3VPB	24	2-D	5	0.9940	0.48	1.000
3VPB	24	3-D	1	0.9939	0.49	1.000
3VPB	24	4-D	3	0.9945	0.46	1.000
3VPB	24	5-D	2	0.9920	0.56	1.000
3VPD	24	1-A	5	0.9824	0.88	1.000
3VPD	24	2-A	4	0.9824	0.88	1.000
3VPD	24	3-A	1	0.9807	0.91	1.000
3VPD	24	4-A	2	0.9829	0.86	1.000
3VPD	24	5-A	3	0.9812	0.91	1.000
3VPD	24	1-B	5	0.9874	0.71	1.000
3VPD	24	2-B	4	0.9881	0.70	1.000

PDB ID:	Recycle Number:	Model Number and PDB Reference Chain:	Rank:	TMscore:	RMSD (Å):	Sequence Identity:
3VPD	24	3-B	1	0.9878	0.72	1.000
3VPD	24	4-B	2	0.9883	0.70	1.000
3VPD	24	5-B	3	0.9866	0.74	1.000
5IG8	24	1-A	5	0.8856	3.78	1.000
5IG8	24	2-A	3	0.8913	3.35	1.000
5IG8	24	3-A	1	0.8832	3.95	1.000
5IG8	24	4-A	4	0.8796	3.97	1.000
5IG8	24	5-A	2	0.8876	3.65	1.000
5IG8	24	1-B	5	0.9814	0.81	1.000
5IG8	24	2-B	3	0.9814	0.81	1.000
5IG8	24	3-B	1	0.9814	0.81	1.000
5IG8	24	4-B	4	0.9789	0.86	1.000
5IG8	24	5-B	2	0.9810	0.82	1.000
5IG9	24	1-A	5	0.9339	4.04	0.981
5IG9	24	2-A	4	0.9459	3.94	0.981
5IG9	24	3-A	2	0.9432	3.96	0.981
5IG9	24	4-A	3	0.9365	4.04	0.981
5IG9	24	5-A	1	0.9470	3.95	0.981
5IG9	24	1-B	5	0.9604	1.46	0.980
5IG9	24	2-B	4	0.9715	1.23	0.980
5IG9	24	3-B	2	0.9713	1.26	0.980
5IG9	24	4-B	3	0.9637	1.42	0.980
5IG9	24	5-B	1	0.9756	1.15	0.980
5IG9	24	1-C	5	0.9451	3.34	0.984
5IG9	24	2-C	4	0.9558	3.25	0.984
5IG9	24	3-C	2	0.9545	3.26	0.984
5IG9	24	4-C	3	0.9498	3.31	0.984
5IG9	24	5-C	1	0.9574	3.26	0.984
5IG9	24	1-D	5	0.9653	1.39	0.984
5IG9	24	2-D	4	0.9764	1.16	0.984
5IG9	24	3-D	2	0.9760	1.18	0.984
5IG9	24	4-D	3	0.9688	1.32	0.984
5IG9	24	5-D	1	0.9796	1.10	0.984
5IG9	24	1-E	5	0.9408	3.77	0.984
5IG9	24	2-E	4	0.9518	3.68	0.984
5IG9	24	3-E	2	0.9504	3.69	0.984
5IG9	24	4-E	3	0.9458	3.74	0.984
5IG9	24	5-E	1	0.9533	3.69	0.984
5IG9	24	1-F	5	0.9681	1.37	0.983
5IG9	24	2-F	4	0.9787	1.16	0.983
5IG9	24	3-F	2	0.9786	1.17	0.983
5IG9	24	4-F	3	0.9728	1.28	0.983
5IG9	24	5-F	1	0.9818	1.10	0.983
5IG9	24	1-G	5	0.9433	3.76	0.984
5IG9	24	2-G	4	0.9532	3.68	0.984
5IG9	24	3-G	2	0.9525	3.68	0.984
5IG9	24	4-G	3	0.9478	3.74	0.984
5IG9	24	5-G	1	0.9554	3.68	0.984
5IG9	24	1-H	5	0.9590	1.39	0.981
5IG9	24	2-H	4	0.9691	1.16	0.981
5IG9	24	3-H	2	0.9697	1.16	0.981
5IG9	24	4-H	3	0.9624	1.34	0.981
5IG9	24	5-H	1	0.9742	1.04	0.981
7DRM	24	1-A	4	0.9260	2.73	1.000
7DRM	24	2-A	3	0.9373	2.57	1.000
7DRM	24	3-A	1	0.9442	2.51	1.000
7DRM	24	4-A	2	0.9238	2.80	1.000
7DRM	24	5-A	5	0.9093	2.86	1.000
7DRM	24	1-B	4	0.9517	1.60	1.000
7DRM	24	2-B	3	0.9546	1.53	1.000
7DRM	24	3-B	1	0.9566	1.52	1.000
7DRM	24	4-B	2	0.9421	1.86	1.000
7DRM	24	5-B	5	0.9417	1.80	1.000
7DRM	24	1-C	4	0.9240	2.72	1.000

PDB ID:	Recycle Number:	Model Number and PDB Reference Chain:	Rank:	TMscore:	RMSD (Å):	Sequence Identity:
7DRM	24	2-C	3	0.9352	2.57	1.000
7DRM	24	3-C	1	0.9428	2.50	1.000
7DRM	24	4-C	2	0.9224	2.77	1.000
7DRM	24	5-C	5	0.9071	2.86	1.000
7DRM	24	1-D	4	0.9188	3.02	0.994
7DRM	24	2-D	3	0.9231	2.93	0.994
7DRM	24	3-D	1	0.9259	2.93	0.994
7DRM	24	4-D	2	0.9099	3.15	0.994
7DRM	24	5-D	5	0.9079	3.11	0.994
7M4S	24	1-A	5	0.9179	2.87	1.000
7M4S	24	2-A	2	0.9162	2.88	1.000
7M4S	24	3-A	1	0.9192	2.88	1.000
7M4S	24	4-A	3	0.9168	2.87	1.000
7M4S	24	5-A	4	0.9183	2.76	1.000
7M4S	24	1-B	5	0.9204	2.60	1.000
7M4S	24	2-B	2	0.9181	2.65	1.000
7M4S	24	3-B	1	0.9207	2.61	1.000
7M4S	24	4-B	3	0.9168	2.67	1.000
7M4S	24	5-B	4	0.9202	2.53	1.000
7M4S	24	1-C	5	0.9225	3.54	1.000
7M4S	24	2-C	2	0.9226	3.53	1.000
7M4S	24	3-C	1	0.9232	3.48	1.000
7M4S	24	4-C	3	0.9202	3.56	1.000
7M4S	24	5-C	4	0.9186	3.57	1.000
7M4S	24	1-D	5	0.9320	4.03	1.000
7M4S	24	2-D	2	0.9323	4.02	1.000
7M4S	24	3-D	1	0.9336	3.97	1.000
7M4S	24	4-D	3	0.9302	4.03	1.000
7M4S	24	5-D	4	0.9319	4.01	1.000
7MGV	24	1-A	3	0.9235	4.15	1.000
7MGV	24	2-A	5	0.9204	4.19	1.000
7MGV	24	3-A	1	0.9254	4.08	1.000
7MGV	24	4-A	4	0.9247	4.29	1.000
7MGV	24	5-A	2	0.9280	4.04	1.000
7MGV	24	1-B	3	0.9796	0.99	1.000
7MGV	24	2-B	5	0.9765	1.06	1.000
7MGV	24	3-B	1	0.9822	0.92	1.000
7MGV	24	4-B	4	0.9622	1.62	1.000
7MGV	24	5-B	2	0.9833	0.89	1.000
3VPB	48	1-A	3	0.9903	0.62	1.000
3VPB	48	2-A	5	0.9901	0.62	1.000
3VPB	48	3-A	1	0.9914	0.58	1.000
3VPB	48	4-A	4	0.9899	0.63	1.000
3VPB	48	5-A	2	0.9899	0.63	1.000
3VPB	48	1-B	3	0.9897	0.64	1.000
3VPB	48	2-B	5	0.9895	0.65	1.000
3VPB	48	3-B	1	0.9907	0.61	1.000
3VPB	48	4-B	4	0.9892	0.66	1.000
3VPB	48	5-B	2	0.9892	0.66	1.000
3VPB	48	1-C	3	0.9933	0.51	1.000
3VPB	48	2-C	5	0.9946	0.46	1.000
3VPB	48	3-C	1	0.9936	0.50	1.000
3VPB	48	4-C	4	0.9945	0.47	1.000
3VPB	48	5-C	2	0.9932	0.52	1.000
3VPB	48	1-D	3	0.9935	0.51	1.000
3VPB	48	2-D	5	0.9948	0.45	1.000
3VPB	48	3-D	1	0.9939	0.49	1.000
3VPB	48	4-D	4	0.9945	0.46	1.000
3VPB	48	5-D	2	0.9934	0.51	1.000
3VPD	48	1-A	5	0.9813	0.90	1.000
3VPD	48	2-A	4	0.9815	0.90	1.000
3VPD	48	3-A	1	0.9796	0.94	1.000
3VPD	48	4-A	2	0.9823	0.88	1.000
3VPD	48	5-A	3	0.9811	0.91	1.000

PDB ID:	Recycle Number:	Model Number and PDB Reference Chain:	Rank:	TMscore:	RMSD (Å):	Sequence Identity:
3VPD	48	1-B	5	0.9869	0.73	1.000
3VPD	48	2-B	4	0.9886	0.68	1.000
3VPD	48	3-B	1	0.9872	0.73	1.000
3VPD	48	4-B	2	0.9879	0.71	1.000
3VPD	48	5-B	3	0.9861	0.75	1.000
5IG8	48	1-A	5	0.8862	3.55	1.000
5IG8	48	2-A	2	0.8913	3.35	1.000
5IG8	48	3-A	1	0.8829	3.99	1.000
5IG8	48	4-A	4	0.8816	3.95	1.000
5IG8	48	5-A	3	0.8854	3.79	1.000
5IG8	48	1-B	5	0.9811	0.82	1.000
5IG8	48	2-B	2	0.9830	0.77	1.000
5IG8	48	3-B	1	0.9820	0.80	1.000
5IG8	48	4-B	4	0.9787	0.87	1.000
5IG8	48	5-B	3	0.9810	0.82	1.000
5IG9	48	1-A	5	0.9381	4.01	0.981
5IG9	48	2-A	4	0.9471	3.93	0.981
5IG9	48	3-A	1	0.9459	3.93	0.981
5IG9	48	4-A	3	0.9383	4.02	0.981
5IG9	48	5-A	2	0.9472	3.96	0.981
5IG9	48	1-B	5	0.9642	1.38	0.980
5IG9	48	2-B	4	0.9727	1.21	0.980
5IG9	48	3-B	1	0.9742	1.19	0.980
5IG9	48	4-B	3	0.9645	1.39	0.980
5IG9	48	5-B	2	0.9753	1.15	0.980
5IG9	48	1-C	5	0.9481	3.33	0.984
5IG9	48	2-C	4	0.9565	3.24	0.984
5IG9	48	3-C	1	0.9563	3.24	0.984
5IG9	48	4-C	3	0.9510	3.31	0.984
5IG9	48	5-C	2	0.9573	3.28	0.984
5IG9	48	1-D	5	0.9688	1.32	0.984
5IG9	48	2-D	4	0.9773	1.15	0.984
5IG9	48	3-D	1	0.9782	1.13	0.984
5IG9	48	4-D	3	0.9697	1.30	0.984
5IG9	48	5-D	2	0.9797	1.08	0.984
5IG9	48	1-E	5	0.9437	3.75	0.984
5IG9	48	2-E	4	0.9524	3.67	0.984
5IG9	48	3-E	1	0.9520	3.66	0.984
5IG9	48	4-E	3	0.9471	3.73	0.984
5IG9	48	5-E	2	0.9532	3.70	0.984
5IG9	48	1-F	5	0.9712	1.31	0.983
5IG9	48	2-F	4	0.9794	1.14	0.983
5IG9	48	3-F	1	0.9804	1.13	0.983
5IG9	48	4-F	3	0.9734	1.27	0.983
5IG9	48	5-F	2	0.9817	1.09	0.983
5IG9	48	1-G	5	0.9461	3.74	0.984
5IG9	48	2-G	4	0.9538	3.67	0.984
5IG9	48	3-G	1	0.9543	3.66	0.984
5IG9	48	4-G	3	0.9488	3.73	0.984
5IG9	48	5-G	2	0.9553	3.70	0.984
5IG9	48	1-H	5	0.9626	1.31	0.981
5IG9	48	2-H	4	0.9703	1.13	0.981
5IG9	48	3-H	1	0.9723	1.09	0.981
5IG9	48	4-H	3	0.9631	1.32	0.981
5IG9	48	5-H	2	0.9743	1.03	0.981
7DRM	48	1-A	5	0.9309	2.67	1.000
7DRM	48	2-A	4	0.9377	2.56	1.000
7DRM	48	3-A	1	0.9447	2.51	1.000
7DRM	48	4-A	2	0.9288	2.63	1.000
7DRM	48	5-A	3	0.9357	2.51	1.000
7DRM	48	1-B	5	0.9458	1.70	1.000
7DRM	48	2-B	4	0.9478	1.64	1.000
7DRM	48	3-B	1	0.9549	1.57	1.000
7DRM	48	4-B	2	0.9469	1.61	1.000

PDB ID:	Recycle Number:	Model Number and PDB Reference Chain:	Rank:	TMscore:	RMSD (Å):	Sequence Identity:
7DRM	48	5-B	3	0.9559	1.46	1.000
7DRM	48	1-C	5	0.9288	2.67	1.000
7DRM	48	2-C	4	0.9359	2.55	1.000
7DRM	48	3-C	1	0.9432	2.49	1.000
7DRM	48	4-C	2	0.9263	2.64	1.000
7DRM	48	5-C	3	0.9326	2.53	1.000
7DRM	48	1-D	5	0.9147	3.06	0.994
7DRM	48	2-D	4	0.9153	3.16	0.994
7DRM	48	3-D	1	0.9236	2.98	0.994
7DRM	48	4-D	2	0.9141	3.02	0.994
7DRM	48	5-D	3	0.9256	2.85	0.994
7M4S	48	1-A	5	0.9186	2.85	1.000
7M4S	48	2-A	2	0.9149	2.91	1.000
7M4S	48	3-A	1	0.9214	2.82	1.000
7M4S	48	4-A	3	0.9206	2.85	1.000
7M4S	48	5-A	4	0.9200	2.70	1.000
7M4S	48	1-B	5	0.9202	2.60	1.000
7M4S	48	2-B	2	0.9172	2.66	1.000
7M4S	48	3-B	1	0.9225	2.56	1.000
7M4S	48	4-B	3	0.9200	2.64	1.000
7M4S	48	5-B	4	0.9215	2.50	1.000
7M4S	48	1-C	5	0.9234	3.50	1.000
7M4S	48	2-C	2	0.9217	3.52	1.000
7M4S	48	3-C	1	0.9244	3.46	1.000
7M4S	48	4-C	3	0.9221	3.56	1.000
7M4S	48	5-C	4	0.9188	3.58	1.000
7M4S	48	1-D	5	0.9335	3.99	1.000
7M4S	48	2-D	2	0.9310	4.01	1.000
7M4S	48	3-D	1	0.9356	3.94	1.000
7M4S	48	4-D	3	0.9341	4.02	1.000
7M4S	48	5-D	4	0.9326	4.02	1.000
7MGV	48	1-A	2	0.9226	4.13	1.000
7MGV	48	2-A	5	0.9198	4.19	1.000
7MGV	48	3-A	1	0.9256	4.06	1.000
7MGV	48	4-A	4	0.9241	4.28	1.000
7MGV	48	5-A	3	0.9290	3.98	1.000
7MGV	48	1-B	2	0.9772	1.05	1.000
7MGV	48	2-B	5	0.9755	1.09	1.000
7MGV	48	3-B	1	0.9818	0.93	1.000
7MGV	48	4-B	4	0.9610	1.63	1.000
7MGV	48	5-B	3	0.9828	0.90	1.000

Table S2. Monomer ATP Grasp Ligase RiPP biosynthetic enzyme US-align results with template. Enzymes with multiple available reference subunits are denoted with their corresponding chain letters.

PDB ID:	Recycle Number:	Model Number and PDB Reference Chain:	Rank:	TMscore:	RMSD (Å):	Sequence Identity:
3VPB	3	1-A	3	0.9906	0.61	1.000
3VPB	3	2-A	4	0.9912	0.59	1.000
3VPB	3	3-A	1	0.9918	0.57	1.000
3VPB	3	4-A	5	0.9885	0.67	1.000
3VPB	3	5-A	2	0.9896	0.64	1.000
3VPB	3	1-B	3	0.9900	0.64	1.000
3VPB	3	2-B	4	0.9906	0.61	1.000
3VPB	3	3-B	1	0.9911	0.59	1.000
3VPB	3	4-B	5	0.9879	0.70	1.000
3VPB	3	5-B	2	0.9889	0.67	1.000
3VPB	3	1-C	3	0.9925	0.54	1.000
3VPB	3	2-C	4	0.9935	0.50	1.000
3VPB	3	3-C	1	0.9939	0.49	1.000
3VPB	3	4-C	5	0.9939	0.49	1.000

PDB ID:	Recycle Number:	Model Number and PDB Reference Chain:	Rank:	TMscore:	RMSD (Å):	Sequence Identity:
3VPB	3	5-C	2	0.9917	0.57	1.000
3VPB	3	1-D	3	0.9928	0.53	1.000
3VPB	3	2-D	4	0.9938	0.49	1.000
3VPB	3	3-D	1	0.9942	0.48	1.000
3VPB	3	4-D	5	0.9940	0.48	1.000
3VPB	3	5-D	2	0.9922	0.56	1.000
3VPD	3	1-A	3	0.9939	0.53	1.000
3VPD	3	2-A	1	0.9931	0.57	1.000
3VPD	3	3-A	2	0.9882	0.88	1.000
3VPD	3	4-A	4	0.9833	0.85	1.000
3VPD	3	5-A	5	0.9814	0.90	1.000
3VPD	3	1-B	3	0.9895	0.65	1.000
3VPD	3	2-B	1	0.9890	0.67	1.000
3VPD	3	3-B	2	0.9882	0.71	1.000
3VPD	3	4-B	4	0.9876	0.72	1.000
3VPD	3	5-B	5	0.9857	0.75	1.000
5IG8	3	1-A	5	0.9197	3.04	1.000
5IG8	3	2-A	3	0.9222	2.87	1.000
5IG8	3	3-A	1	0.8869	3.55	1.000
5IG8	3	4-A	4	0.8808	3.97	1.000
5IG8	3	5-A	1	0.9197	3.04	1.000
5IG8	3	1-B	5	0.9919	0.54	1.000
5IG8	3	2-B	3	0.9913	0.56	1.000
5IG8	3	3-B	1	0.9818	0.80	1.000
5IG8	3	4-B	4	0.9813	0.81	1.000
5IG8	3	5-B	2	0.9801	0.84	1.000
5IG9	3	1-A	2	0.9583	3.89	0.981
5IG9	3	2-A	1	0.9583	3.89	0.981
5IG9	3	3-A	3	0.9450	3.94	0.981
5IG9	3	4-A	5	0.9411	3.96	0.981
5IG9	3	5-A	4	0.9465	3.94	0.981
5IG9	3	1-B	2	0.9885	0.89	0.981
5IG9	3	2-B	1	0.9888	0.88	0.981
5IG9	3	3-B	3	0.9726	1.22	0.981
5IG9	3	4-B	5	0.9678	1.32	0.981
5IG9	3	5-B	4	0.9749	1.17	0.981
5IG9	3	1-C	2	0.9674	3.17	0.984
5IG9	3	2-C	1	0.9669	3.19	0.984
5IG9	3	3-C	3	0.9552	3.25	0.984
5IG9	3	4-C	5	0.9528	3.25	0.984
5IG9	3	5-C	4	0.9563	3.25	0.984
5IG9	3	1-D	2	0.9901	0.87	0.984
5IG9	3	2-D	1	0.9899	0.88	0.984
5IG9	3	3-D	3	0.9771	1.15	0.984
5IG9	3	4-D	5	0.9728	1.23	0.984
5IG9	3	5-D	4	0.9780	1.14	0.984
5IG9	3	1-E	2	0.9634	3.63	0.984
5IG9	3	2-E	1	0.9627	3.64	0.984
5IG9	3	3-E	3	0.9511	3.68	0.984
5IG9	3	4-E	5	0.9487	3.69	0.984
5IG9	3	5-E	4	0.9521	3.68	0.984
5IG9	3	1-F	2	0.9923	0.88	0.983
5IG9	3	2-F	1	0.9921	0.88	0.983
5IG9	3	3-F	3	0.9791	1.16	0.983
5IG9	3	4-F	5	0.9760	1.21	0.983
5IG9	3	5-F	4	0.9800	1.14	0.983
5IG9	3	1-G	2	0.9648	3.63	0.984
5IG9	3	2-G	1	0.9642	3.64	0.984
5IG9	3	3-G	3	0.9530	3.67	0.984
5IG9	3	4-G	5	0.9507	3.68	0.984
5IG9	3	5-G	4	0.9543	3.67	0.984
5IG9	3	1-H	2	0.9868	0.73	0.981
5IG9	3	2-H	1	0.9874	0.69	0.981
5IG9	3	3-H	3	0.9710	1.12	0.981

PDB ID:	Recycle Number:	Model Number and PDB Reference Chain:	Rank:	TMscore:	RMSD (Å):	Sequence Identity:
5IG9	3	4-H	5	0.9654	1.27	0.981
5IG9	3	5-H	4	0.9729	1.07	0.981
7DRM	3	1-A	5	0.9451	2.68	1.000
7DRM	3	2-A	2	0.9494	2.45	1.000
7DRM	3	3-A	1	0.9474	2.44	1.000
7DRM	3	4-A	3	0.9311	2.68	1.000
7DRM	3	5-A	4	0.9242	2.68	1.000
7DRM	3	1-B	5	0.9557	1.52	1.000
7DRM	3	2-B	2	0.9516	1.59	1.000
7DRM	3	3-B	1	0.9559	1.51	1.000
7DRM	3	4-B	3	0.9464	1.75	1.000
7DRM	3	5-B	4	0.9501	1.65	1.000
7DRM	3	1-C	5	0.9436	2.51	1.000
7DRM	3	2-C	2	0.9480	2.45	1.000
7DRM	3	3-C	1	0.9461	2.43	1.000
7DRM	3	4-C	3	0.9296	2.66	1.000
7DRM	3	5-C	4	0.9220	2.68	1.000
7DRM	3	1-D	5	0.9263	2.91	0.994
7DRM	3	2-D	2	0.9233	2.93	0.994
7DRM	3	3-D	1	0.9261	2.90	0.994
7DRM	3	4-D	3	0.9150	3.07	0.994
7DRM	3	5-D	4	0.9173	2.99	0.994
7M4S	3	1-A	5	0.9177	2.87	1.000
7M4S	3	2-A	2	0.9216	2.80	1.000
7M4S	3	3-A	1	0.9177	2.83	1.000
7M4S	3	4-A	3	0.9115	2.94	1.000
7M4S	3	5-A	4	0.9116	2.98	1.000
7M4S	3	1-B	5	0.9221	2.63	1.000
7M4S	3	2-B	2	0.9256	2.56	1.000
7M4S	3	3-B	1	0.9166	2.64	1.000
7M4S	3	4-B	3	0.9113	2.75	1.000
7M4S	3	5-B	4	0.9108	2.75	1.000
7M4S	3	1-C	5	0.9194	3.57	1.000
7M4S	3	2-C	2	0.9234	3.53	1.000
7M4S	3	3-C	1	0.9220	3.44	1.000
7M4S	3	4-C	3	0.9173	3.54	1.000
7M4S	3	5-C	4	0.9154	3.58	1.000
7M4S	3	1-D	5	0.9311	4.02	1.000
7M4S	3	2-D	2	0.9368	3.98	1.000
7M4S	3	3-D	1	0.9335	3.91	1.000
7M4S	3	4-D	3	0.9274	4.00	1.000
7M4S	3	5-D	4	0.9272	4.03	1.000
7MGV	3	1-A	5	0.9265	4.11	1.000
7MGV	3	2-A	3	0.9195	4.21	1.000
7MGV	3	3-A	1	0.9247	4.08	1.000
7MGV	3	4-A	4	0.9229	4.31	1.000
7MGV	3	5-A	2	0.9245	4.13	1.000
7MGV	3	1-B	5	0.9827	0.91	1.000
7MGV	3	2-B	3	0.9755	1.09	1.000
7MGV	3	3-B	1	0.9813	0.94	1.000
7MGV	3	4-B	4	0.9619	1.58	1.000
7MGV	3	5-B	2	0.9818	0.93	1.000
3VPB	12	1-A	3	0.9907	0.61	1.000
3VPB	12	2-A	5	0.9911	0.59	1.000
3VPB	12	3-A	1	0.9907	0.61	1.000
3VPB	12	4-A	4	0.9901	0.63	1.000
3VPB	12	5-A	2	0.9886	0.68	1.000
3VPB	12	1-B	3	0.9900	0.63	1.000
3VPB	12	2-B	5	0.9905	0.62	1.000
3VPB	12	3-B	1	0.9900	0.63	1.000
3VPB	12	4-B	4	0.9895	0.65	1.000
3VPB	12	5-B	2	0.9880	0.70	1.000
3VPB	12	1-C	3	0.9929	0.53	1.000
3VPB	12	2-C	5	0.9939	0.49	1.000

PDB ID:	Recycle Number:	Model Number and PDB Reference Chain:	Rank:	TMscore:	RMSD (Å):	Sequence Identity:
3VPB	12	3-C	1	0.9935	0.50	1.000
3VPB	12	4-C	4	0.9944	0.47	1.000
3VPB	12	5-C	2	0.9916	0.58	1.000
3VPB	12	1-D	3	0.9932	0.52	1.000
3VPB	12	2-D	5	0.9918	0.57	1.000
3VPB	12	3-D	1	0.9939	0.49	1.000
3VPB	12	4-D	4	0.9946	0.46	1.000
3VPB	12	5-D	2	0.9918	0.57	1.000
3VPD	12	1-A	3	0.9940	0.53	1.000
3VPD	12	2-A	1	0.9930	0.58	1.000
3VPD	12	3-A	2	0.9802	0.93	1.000
3VPD	12	4-A	4	0.9829	0.86	1.000
3VPD	12	5-A	5	0.9806	0.92	1.000
3VPD	12	1-B	3	0.9898	0.65	1.000
3VPD	12	2-B	1	0.9893	0.67	1.000
3VPD	12	3-B	2	0.9878	0.72	1.000
3VPD	12	4-B	4	0.9881	0.71	1.000
3VPD	12	5-B	5	0.9864	0.74	1.000
5IG8	12	1-A	5	0.9197	3.04	1.000
5IG8	12	2-A	4	0.9231	2.81	1.000
5IG8	12	3-A	1	0.8831	3.91	1.000
5IG8	12	4-A	3	0.8816	3.94	1.000
5IG8	12	5-A	2	0.8868	3.72	1.000
5IG8	12	1-B	5	0.9921	0.54	1.000
5IG8	12	2-B	4	0.9916	0.55	1.000
5IG8	12	3-B	1	0.9813	0.81	1.000
5IG8	12	4-B	3	0.9798	0.84	1.000
5IG8	12	5-B	2	0.9921	0.54	1.000
5IG9	12	1-A	2	0.9601	3.87	0.981
5IG9	12	2-A	1	0.9601	3.87	0.981
5IG9	12	3-A	3	0.9431	3.96	0.981
5IG9	12	4-A	5	0.9369	4.03	0.981
5IG9	12	5-A	4	0.9481	3.94	0.981
5IG9	12	1-B	2	0.9904	0.82	0.980
5IG9	12	2-B	1	0.9910	0.81	0.980
5IG9	12	3-B	3	0.9709	1.26	0.980
5IG9	12	4-B	5	0.9639	1.41	0.980
5IG9	12	5-B	4	0.9764	1.13	0.980
5IG9	12	1-C	2	0.9692	3.17	0.984
5IG9	12	2-C	1	0.9693	3.16	0.984
5IG9	12	3-C	3	0.9540	3.26	0.984
5IG9	12	4-C	5	0.9501	3.31	0.984
5IG9	12	5-C	4	0.9577	3.25	0.984
5IG9	12	1-D	2	0.9921	0.80	0.984
5IG9	12	2-D	1	0.9920	0.81	0.984
5IG9	12	3-D	3	0.9756	1.19	0.984
5IG9	12	4-D	5	0.9691	1.31	0.984
5IG9	12	5-D	4	0.9801	1.08	0.984
5IG9	12	1-E	2	0.9651	3.62	0.984
5IG9	12	2-E	1	0.9651	3.61	0.984
5IG9	12	3-E	3	0.9499	3.69	0.984
5IG9	12	4-E	5	0.9463	3.74	0.984
5IG9	12	5-E	4	0.9537	3.68	0.984
5IG9	12	1-F	2	0.9943	0.81	0.983
5IG9	12	2-F	1	0.9943	0.81	0.983
5IG9	12	3-F	3	0.9780	1.18	0.983
5IG9	12	4-F	5	0.9731	1.27	0.983
5IG9	12	5-F	4	0.9820	1.09	0.983
5IG9	12	1-G	2	0.9665	3.61	0.984
5IG9	12	2-G	1	0.9667	3.61	0.984
5IG9	12	3-G	3	0.9519	3.69	0.984
5IG9	12	4-G	5	0.9481	3.73	0.984
5IG9	12	5-G	4	0.9557	3.68	0.984
5IG9	12	1-H	2	0.9889	0.63	0.981

PDB ID:	Recycle Number:	Model Number and PDB Reference Chain:	Rank:	TMscore:	RMSD (Å):	Sequence Identity:
5IG9	12	2-H	1	0.9896	0.59	0.981
5IG9	12	3-H	3	0.9693	1.16	0.981
5IG9	12	4-H	5	0.9625	1.34	0.981
5IG9	12	5-H	4	0.9748	1.02	0.981
7DRM	12	1-A	5	0.9432	2.56	1.000
7DRM	12	2-A	3	0.9476	2.48	1.000
7DRM	12	3-A	1	0.9445	2.49	1.000
7DRM	12	4-A	2	0.9264	2.77	1.000
7DRM	12	5-A	4	0.9166	2.77	1.000
7DRM	12	1-B	5	0.9536	1.56	1.000
7DRM	12	2-B	3	0.9492	1.62	1.000
7DRM	12	3-B	1	0.9567	1.51	1.000
7DRM	12	4-B	2	0.9422	1.86	1.000
7DRM	12	5-B	4	0.9454	1.73	1.000
7DRM	12	1-C	5	0.9416	2.55	1.000
7DRM	12	2-C	3	0.9464	2.47	1.000
7DRM	12	3-C	1	0.9464	2.47	1.000
7DRM	12	4-C	2	0.9431	2.48	1.000
7DRM	12	5-C	4	0.9249	2.73	1.000
7DRM	12	1-D	5	0.9239	2.96	0.994
7DRM	12	2-D	3	0.9210	2.96	0.994
7DRM	12	3-D	1	0.9260	2.92	0.994
7DRM	12	4-D	2	0.9104	3.15	0.994
7DRM	12	5-D	4	0.9122	3.06	0.994
7M4S	12	1-A	4	0.9164	2.92	1.000
7M4S	12	2-A	1	0.9209	2.82	1.000
7M4S	12	3-A	2	0.9187	2.84	1.000
7M4S	12	4-A	3	0.9169	2.83	1.000
7M4S	12	5-A	5	0.9189	2.78	1.000
7M4S	12	1-B	4	0.9208	2.65	1.000
7M4S	12	2-B	1	0.9239	2.59	1.000
7M4S	12	3-B	2	0.9196	2.59	1.000
7M4S	12	4-B	3	0.9162	2.65	1.000
7M4S	12	5-B	5	0.9198	2.55	1.000
7M4S	12	1-C	4	0.9192	3.56	1.000
7M4S	12	2-C	1	0.9228	3.52	1.000
7M4S	12	3-C	2	0.9227	3.46	1.000
7M4S	12	4-C	3	0.9205	3.53	1.000
7M4S	12	5-C	5	0.9198	3.56	1.000
7M4S	12	1-D	4	0.9302	4.02	1.000
7M4S	12	2-D	1	0.9354	3.97	1.000
7M4S	12	3-D	2	0.9333	3.94	1.000
7M4S	12	4-D	3	0.9304	4.01	1.000
7M4S	12	5-D	5	0.9331	4.01	1.000
7MGV	12	1-A	4	0.9250	4.13	1.000
7MGV	12	2-A	5	0.9191	4.23	1.000
7MGV	12	3-A	1	0.9247	4.09	1.000
7MGV	12	4-A	3	0.9245	4.28	1.000
7MGV	12	5-A	2	0.9264	4.12	1.000
7MGV	12	1-B	4	0.9806	0.96	1.000
7MGV	12	2-B	5	0.9747	1.11	1.000
7MGV	12	3-B	1	0.9814	0.94	1.000
7MGV	12	4-B	3	0.9618	1.63	1.000
7MGV	12	5-B	2	0.9838	0.87	1.000
3VPB	24	1-A	3	0.9904	0.62	1.000
3VPB	24	2-A	5	0.9910	0.60	1.000
3VPB	24	3-A	1	0.9914	0.58	1.000
3VPB	24	4-A	4	0.9903	0.62	1.000
3VPB	24	5-A	2	0.9886	0.67	1.000
3VPB	24	1-B	3	0.9898	0.64	1.000
3VPB	24	2-B	5	0.9904	0.62	1.000
3VPB	24	3-B	1	0.9898	0.64	1.000
3VPB	24	4-B	4	0.9898	0.64	1.000
3VPB	24	5-B	2	0.9881	0.70	1.000

PDB ID:	Recycle Number:	Model Number and PDB Reference Chain:	Rank:	TMscore:	RMSD (Å):	Sequence Identity:
3VPB	24	1-C	3	0.9928	0.53	1.000
3VPB	24	2-C	5	0.9939	0.49	1.000
3VPB	24	3-C	1	0.9936	0.50	1.000
3VPB	24	4-C	4	0.9944	0.47	1.000
3VPB	24	5-C	2	0.9918	0.57	1.000
3VPB	24	1-D	3	0.9931	0.52	1.000
3VPB	24	2-D	5	0.9942	0.48	1.000
3VPB	24	3-D	1	0.9939	0.49	1.000
3VPB	24	4-D	4	0.9945	0.46	1.000
3VPB	24	5-D	2	0.9920	0.56	1.000
3VPD	24	1-A	3	0.9940	0.53	1.000
3VPD	24	2-A	1	0.9930	0.58	1.000
3VPD	24	3-A	2	0.9807	0.91	1.000
3VPD	24	4-A	4	0.9829	0.86	1.000
3VPD	24	5-A	5	0.9812	0.90	1.000
3VPD	24	1-B	3	0.9896	0.65	1.000
3VPD	24	2-B	1	0.9893	0.67	1.000
3VPD	24	3-B	2	0.9878	0.72	1.000
3VPD	24	4-B	4	0.9883	0.70	1.000
3VPD	24	5-B	5	0.9866	0.74	1.000
5IG8	24	1-A	5	0.9201	3.00	1.000
5IG8	24	2-A	4	0.9234	2.81	1.000
5IG8	24	3-A	1	0.8832	3.95	1.000
5IG8	24	4-A	3	0.8796	3.97	1.000
5IG8	24	5-A	2	0.8876	3.65	1.000
5IG8	24	1-B	5	0.9921	0.54	1.000
5IG8	24	2-B	4	0.9918	0.54	1.000
5IG8	24	3-B	1	0.9814	0.81	1.000
5IG8	24	4-B	3	0.9788	0.86	1.000
5IG8	24	5-B	2	0.9810	0.82	1.000
5IG9	24	1-A	2	0.9602	3.87	0.981
5IG9	24	2-A	1	0.9602	3.87	0.981
5IG9	24	3-A	4	0.9432	3.96	0.981
5IG9	24	4-A	5	0.9365	4.04	0.981
5IG9	24	5-A	3	0.9470	3.95	0.981
5IG9	24	1-B	2	0.9906	0.82	0.980
5IG9	24	2-B	1	0.9912	0.80	0.980
5IG9	24	3-B	4	0.9713	1.26	0.980
5IG9	24	4-B	5	0.9637	1.42	0.980
5IG9	24	5-B	3	0.9756	1.15	0.980
5IG9	24	1-C	2	0.9693	3.16	0.984
5IG9	24	2-C	1	0.9695	3.16	0.984
5IG9	24	3-C	4	0.9545	3.26	0.984
5IG9	24	4-C	5	0.9498	3.31	0.984
5IG9	24	5-C	3	0.9574	3.26	0.984
5IG9	24	1-D	2	0.9922	0.80	0.984
5IG9	24	2-D	1	0.9921	0.81	0.984
5IG9	24	3-D	4	0.9760	1.18	0.984
5IG9	24	4-D	5	0.9688	1.32	0.984
5IG9	24	5-D	3	0.9796	1.10	0.984
5IG9	24	1-E	2	0.9652	3.61	0.984
5IG9	24	2-E	1	0.9652	3.61	0.984
5IG9	24	3-E	4	0.9504	3.69	0.984
5IG9	24	4-E	5	0.9458	3.74	0.984
5IG9	24	5-E	3	0.9533	3.69	0.984
5IG9	24	1-F	2	0.9944	0.81	0.983
5IG9	24	2-F	1	0.9945	0.81	0.983
5IG9	24	3-F	4	0.9786	1.17	0.983
5IG9	24	4-F	5	0.9728	1.28	0.983
5IG9	24	5-F	3	0.9818	1.10	0.983
5IG9	24	1-G	2	0.9666	3.60	0.984
5IG9	24	2-G	1	0.9669	3.61	0.984
5IG9	24	3-G	4	0.9525	3.68	0.984
5IG9	24	4-G	5	0.9478	3.74	0.984

PDB ID:	Recycle Number:	Model Number and PDB Reference Chain:	Rank:	TMscore:	RMSD (Å):	Sequence Identity:
5IG9	24	5-G	3	0.9554	3.68	0.984
5IG9	24	1-H	2	0.9891	0.63	0.981
5IG9	24	2-H	1	0.9895	0.60	0.981
5IG9	24	3-H	4	0.9697	1.16	0.981
5IG9	24	4-H	5	0.9624	1.34	0.981
5IG9	24	5-H	3	0.9742	1.04	0.981
7DRM	24	1-A	4	0.9435	2.55	1.000
7DRM	24	2-A	2	0.9478	2.48	1.000
7DRM	24	3-A	1	0.9442	2.51	1.000
7DRM	24	4-A	3	0.9238	2.80	1.000
7DRM	24	5-A	5	0.9092	2.86	1.000
7DRM	24	1-B	4	0.9548	1.54	1.000
7DRM	24	2-B	2	0.9507	1.59	1.000
7DRM	24	3-B	1	0.9566	1.52	1.000
7DRM	24	4-B	3	0.9421	1.86	1.000
7DRM	24	5-B	5	0.9414	1.80	1.000
7DRM	24	1-C	4	0.9420	2.54	1.000
7DRM	24	2-C	2	0.9466	2.47	1.000
7DRM	24	3-C	1	0.9428	2.50	1.000
7DRM	24	4-C	3	0.9224	2.77	1.000
7DRM	24	5-C	5	0.9070	2.86	1.000
7DRM	24	1-D	4	0.9250	2.94	0.994
7DRM	24	2-D	2	0.9225	2.94	0.994
7DRM	24	3-D	1	0.9259	2.93	0.994
7DRM	24	4-D	3	0.9099	3.15	0.994
7DRM	24	5-D	5	0.9077	3.11	0.994
7M4S	24	1-A	5	0.9161	2.93	1.000
7M4S	24	2-A	1	0.9200	2.83	1.000
7M4S	24	3-A	2	0.9192	2.88	1.000
7M4S	24	4-A	3	0.9168	2.87	1.000
7M4S	24	5-A	4	0.9183	2.76	1.000
7M4S	24	1-B	5	0.9212	2.65	1.000
7M4S	24	2-B	1	0.9240	2.58	1.000
7M4S	24	3-B	2	0.9207	2.61	1.000
7M4S	24	4-B	3	0.9168	2.67	1.000
7M4S	24	5-B	4	0.9202	2.53	1.000
7M4S	24	1-C	5	0.9180	3.59	1.000
7M4S	24	2-C	1	0.9212	3.54	1.000
7M4S	24	3-C	2	0.9232	3.48	1.000
7M4S	24	4-C	3	0.9202	3.56	1.000
7M4S	24	5-C	4	0.9186	3.57	1.000
7M4S	24	1-D	5	0.9291	4.05	1.000
7M4S	24	2-D	1	0.9338	3.99	1.000
7M4S	24	3-D	2	0.9336	3.97	1.000
7M4S	24	4-D	3	0.9302	4.03	1.000
7M4S	24	5-D	4	0.9319	4.01	1.000
7MGV	24	1-A	5	0.9247	4.14	1.000
7MGV	24	2-A	4	0.9177	4.24	1.000
7MGV	24	3-A	1	0.9254	4.08	1.000
7MGV	24	4-A	3	0.9247	4.29	1.000
7MGV	24	5-A	2	0.9280	4.04	1.000
7MGV	24	1-B	5	0.9805	0.97	1.000
7MGV	24	2-B	4	0.9734	1.14	1.000
7MGV	24	3-B	1	0.9822	0.92	1.000
7MGV	24	4-B	3	0.9622	1.62	1.000
7MGV	24	5-B	2	0.9833	0.89	1.000
3VPB	48	1-A	3	0.9919	0.57	1.000
3VPB	48	2-A	1	0.9920	0.56	1.000
3VPB	48	3-A	2	0.9914	0.58	1.000
3VPB	48	4-A	5	0.9899	0.63	1.000
3VPB	48	5-A	4	0.9899	0.63	1.000
3VPB	48	1-B	3	0.9914	0.59	1.000
3VPB	48	2-B	1	0.9913	0.59	1.000
3VPB	48	3-B	2	0.9907	0.61	1.000

PDB ID:	Recycle Number:	Model Number and PDB Reference Chain:	Rank:	TMscore:	RMSD (Å):	Sequence Identity:
3VPB	48	4-B	5	0.9892	0.66	1.000
3VPB	48	5-B	4	0.9892	0.66	1.000
3VPB	48	1-C	3	0.9977	0.30	1.000
3VPB	48	2-C	1	0.9978	0.29	1.000
3VPB	48	3-C	2	0.9936	0.50	1.000
3VPB	48	4-C	5	0.9945	0.47	1.000
3VPB	48	5-C	4	0.9932	0.52	1.000
3VPB	48	1-D	3	0.9977	0.29	1.000
3VPB	48	2-D	1	0.9979	0.29	1.000
3VPB	48	3-D	2	0.9939	0.49	1.000
3VPB	48	4-D	5	0.9945	0.46	1.000
3VPB	48	5-D	4	0.9934	0.51	1.000
3VPD	48	1-A	2	0.9935	0.55	1.000
3VPD	48	2-A	1	0.9927	0.59	1.000
3VPD	48	3-A	3	0.9796	0.94	1.000
3VPD	48	4-A	4	0.9823	0.88	1.000
3VPD	48	5-A	5	0.9811	0.91	1.000
3VPD	48	1-B	2	0.9877	0.71	1.000
3VPD	48	2-B	1	0.9875	0.72	1.000
3VPD	48	3-B	3	0.9872	0.73	1.000
3VPD	48	4-B	4	0.9879	0.71	1.000
3VPD	48	5-B	5	0.9861	0.75	1.000
5IG8	48	1-A	5	0.9135	3.13	1.000
5IG8	48	2-A	2	0.9164	3.03	1.000
5IG8	48	3-A	1	0.8829	3.99	1.000
5IG8	48	4-A	4	0.8816	3.95	1.000
5IG8	48	5-A	3	0.8854	3.79	1.000
5IG8	48	1-B	5	0.9928	0.50	1.000
5IG8	48	2-B	2	0.9920	0.53	1.000
5IG8	48	3-B	1	0.9820	0.80	1.000
5IG8	48	4-B	4	0.9787	0.87	1.000
5IG8	48	5-B	3	0.9810	0.82	1.000
5IG9	48	1-A	2	0.9619	3.80	0.981
5IG9	48	2-A	1	0.9604	3.80	0.981
5IG9	48	3-A	3	0.9459	3.93	0.981
5IG9	48	4-A	5	0.9383	4.02	0.981
5IG9	48	5-A	4	0.9472	3.96	0.981
5IG9	48	1-B	2	0.9900	0.84	0.980
5IG9	48	2-B	1	0.9897	0.85	0.980
5IG9	48	3-B	3	0.9742	1.19	0.980
5IG9	48	4-B	5	0.9645	1.39	0.980
5IG9	48	5-B	4	0.9753	1.15	0.980
5IG9	48	1-C	2	0.9710	3.11	0.984
5IG9	48	2-C	1	0.9699	3.11	0.984
5IG9	48	3-C	3	0.9563	3.24	0.984
5IG9	48	4-C	5	0.9510	3.31	0.984
5IG9	48	5-C	4	0.9573	3.28	0.984
5IG9	48	1-D	2	0.9915	0.83	0.984
5IG9	48	2-D	1	0.9902	0.87	0.984
5IG9	48	3-D	3	0.9782	1.13	0.984
5IG9	48	4-D	5	0.9697	1.30	0.984
5IG9	48	5-D	4	0.9797	1.08	0.984
5IG9	48	1-E	2	0.9667	3.55	0.984
5IG9	48	2-E	1	0.9654	3.55	0.984
5IG9	48	3-E	3	0.9520	3.66	0.984
5IG9	48	4-E	5	0.9471	3.73	0.984
5IG9	48	5-E	4	0.9532	3.70	0.984
5IG9	48	1-F	2	0.9937	0.83	0.983
5IG9	48	2-F	1	0.9927	0.86	0.983
5IG9	48	3-F	3	0.9804	1.13	0.983
5IG9	48	4-F	5	0.9734	1.27	0.983
5IG9	48	5-F	4	0.9817	1.09	0.983
5IG9	48	1-G	2	0.9683	3.54	0.984
5IG9	48	2-G	1	0.9673	3.54	0.984

PDB ID:	Recycle Number:	Model Number and PDB Reference Chain:	Rank:	TMscore:	RMSD (Å):	Sequence Identity:
5IG9	48	3-G	3	0.9543	3.66	0.984
5IG9	48	4-G	5	0.9488	3.73	0.984
5IG9	48	5-G	4	0.9553	3.70	0.984
5IG9	48	1-H	2	0.9881	0.67	0.981
5IG9	48	2-H	1	0.9877	0.68	0.981
5IG9	48	3-H	3	0.9723	1.09	0.981
5IG9	48	4-H	5	0.9631	1.32	0.981
5IG9	48	5-H	4	0.9743	1.03	0.981
7DRM	48	1-A	2	0.9775	2.09	1.000
7DRM	48	2-A	1	0.9774	2.11	1.000
7DRM	48	3-A	3	0.9447	2.51	1.000
7DRM	48	4-A	4	0.9288	2.63	1.000
7DRM	48	5-A	5	0.9357	2.51	1.000
7DRM	48	1-B	2	0.9591	1.46	1.000
7DRM	48	2-B	1	0.9584	1.47	1.000
7DRM	48	3-B	3	0.9549	1.57	1.000
7DRM	48	4-B	4	0.9469	1.61	1.000
7DRM	48	5-B	5	0.9559	1.46	1.000
7DRM	48	1-C	2	0.9760	2.10	1.000
7DRM	48	2-C	1	0.9761	2.12	1.000
7DRM	48	3-C	3	0.9432	2.49	1.000
7DRM	48	4-C	4	0.9263	2.64	1.000
7DRM	48	5-C	5	0.9326	2.53	1.000
7DRM	48	1-D	2	0.9331	2.84	0.994
7DRM	48	2-D	1	0.9324	2.84	0.994
7DRM	48	3-D	3	0.9236	2.98	0.994
7DRM	48	4-D	4	0.9141	3.02	0.994
7DRM	48	5-D	5	0.9256	2.85	0.994
7M4S	48	1-A	3	0.9343	2.67	1.000
7M4S	48	2-A	1	0.9310	2.69	1.000
7M4S	48	3-A	2	0.9214	2.82	1.000
7M4S	48	4-A	4	0.9206	2.85	1.000
7M4S	48	5-A	5	0.9200	2.70	1.000
7M4S	48	1-B	3	0.9409	2.40	1.000
7M4S	48	2-B	1	0.9375	2.42	1.000
7M4S	48	3-B	2	0.9225	2.56	1.000
7M4S	48	4-B	4	0.9200	2.64	1.000
7M4S	48	5-B	5	0.9215	2.50	1.000
7M4S	48	1-C	3	0.9267	3.54	1.000
7M4S	48	2-C	1	0.9261	3.51	1.000
7M4S	48	3-C	2	0.9244	3.46	1.000
7M4S	48	4-C	4	0.9221	3.56	1.000
7M4S	48	5-C	5	0.9188	3.58	1.000
7M4S	48	1-D	3	0.9415	3.97	1.000
7M4S	48	2-D	1	0.9408	3.93	1.000
7M4S	48	3-D	2	0.9356	3.94	1.000
7M4S	48	4-D	4	0.9341	4.02	1.000
7M4S	48	5-D	5	0.9326	4.02	1.000
7MGV	48	1-A	2	0.9355	4.00	1.000
7MGV	48	2-A	1	0.9333	4.02	1.000
7MGV	48	3-A	3	0.9256	4.06	1.000
7MGV	48	4-A	5	0.9241	4.28	1.000
7MGV	48	5-A	4	0.9290	3.98	1.000
7MGV	48	1-B	2	0.9965	0.42	1.000
7MGV	48	2-B	1	0.9948	0.50	1.000
7MGV	48	3-B	3	0.9818	0.93	1.000
7MGV	48	4-B	5	0.9610	1.63	1.000
7MGV	48	5-B	4	0.9828	0.90	1.000

Table S3. Monomer ATP Grasp Ligase RiPP biosynthetic enzyme US-align results with template and AMBER. Enzymes with multiple available reference subunits are denoted with their corresponding chain letters.

PDB ID:	Recycle Number:	Model Number and PDB Reference Chain:	Rank:	TMscore:	RMSD(Å):	Sequence Identity:
3VPB	3	1-A	3	0.9905	0.61	1.000
3VPB	3	2-A	4	0.9911	0.59	1.000
3VPB	3	3-A	1	0.9917	0.57	1.000
3VPB	3	4-A	5	0.9885	0.67	1.000
3VPB	3	5-A	2	0.9895	0.65	1.000
3VPB	3	1-B	3	0.9899	0.64	1.000
3VPB	3	2-B	4	0.9906	0.61	1.000
3VPB	3	3-B	1	0.9911	0.60	1.000
3VPB	3	4-B	5	0.9879	0.70	1.000
3VPB	3	5-B	2	0.9889	0.67	1.000
3VPB	3	1-C	3	0.9924	0.55	1.000
3VPB	3	2-C	4	0.9934	0.51	1.000
3VPB	3	3-C	1	0.9938	0.49	1.000
3VPB	3	4-C	5	0.9939	0.49	1.000
3VPB	3	5-C	2	0.9916	0.57	1.000
3VPB	3	1-D	3	0.9927	0.54	1.000
3VPB	3	2-D	4	0.9937	0.50	1.000
3VPB	3	3-D	1	0.9941	0.48	1.000
3VPB	3	4-D	5	0.9940	0.49	1.000
3VPB	3	5-D	2	0.9921	0.56	1.000
3VPD	3	1-A	3	0.9939	0.54	1.000
3VPD	3	2-A	1	0.9931	0.57	1.000
3VPD	3	3-A	2	0.9821	0.88	1.000
3VPD	3	4-A	4	0.9834	0.85	1.000
3VPD	3	5-A	5	0.9813	0.90	1.000
3VPD	3	1-B	3	0.9895	0.65	1.000
3VPD	3	2-B	1	0.9890	0.67	1.000
3VPD	3	3-B	2	0.9882	0.70	1.000
3VPD	3	4-B	4	0.9878	0.71	1.000
3VPD	3	5-B	5	0.9858	0.76	1.000
5IG8	3	1-A	5	0.9196	3.05	1.000
5IG8	3	2-A	3	0.9219	2.88	1.000
5IG8	3	3-A	1	0.8866	3.56	1.000
5IG8	3	4-A	4	0.8806	3.97	1.000
5IG8	3	5-A	2	0.8856	3.82	1.000
5IG8	3	1-B	5	0.9921	0.54	1.000
5IG8	3	2-B	3	0.9915	0.56	1.000
5IG8	3	3-B	1	0.9818	0.80	1.000
5IG8	3	4-B	4	0.9813	0.81	1.000
5IG8	3	5-B	2	0.9800	0.84	1.000
5IG9	3	1-A	2	0.9584	3.88	0.981
5IG9	3	2-A	1	0.9574	3.90	0.981
5IG9	3	3-A	3	0.9449	3.94	0.981
5IG9	3	4-A	5	0.9411	3.96	0.981
5IG9	3	5-A	4	0.9464	3.95	0.981
5IG9	3	1-B	2	0.9884	0.89	0.980
5IG9	3	2-B	1	0.9886	0.89	0.980
5IG9	3	3-B	3	0.9726	1.22	0.980
5IG9	3	4-B	5	0.9678	1.32	0.980
5IG9	3	5-B	4	0.9748	1.18	0.980
5IG9	3	1-C	2	0.9674	3.17	0.984
5IG9	3	2-C	1	0.9667	3.19	0.984
5IG9	3	3-C	3	0.9551	3.25	0.984
5IG9	3	4-C	5	0.9528	3.25	0.984
5IG9	3	5-C	4	0.9561	3.25	0.984
5IG9	3	1-D	2	0.9900	0.87	0.984
5IG9	3	2-D	1	0.9897	0.88	0.984
5IG9	3	3-D	3	0.9770	1.15	0.984
5IG9	3	4-D	5	0.9729	1.23	0.984
5IG9	3	5-D	4	0.9779	1.14	0.984
5IG9	3	1-E	2	0.9634	3.62	0.984
5IG9	3	2-E	1	0.9625	3.64	0.984
5IG9	3	3-E	3	0.9510	3.68	0.984

PDB ID:	Recycle Number:	Model Number and PDB Reference Chain:	Rank:	TMscore:	RMSD(Å):	Sequence Identity:
5IG9	3	4-E	5	0.9487	3.69	0.984
5IG9	3	5-E	4	0.9520	3.68	0.984
5IG9	3	1-F	2	0.9922	0.88	0.983
5IG9	3	2-F	1	0.9920	0.89	0.983
5IG9	3	3-F	3	0.9791	1.16	0.983
5IG9	3	4-F	5	0.9760	1.21	0.983
5IG9	3	5-F	4	0.9800	1.14	0.983
5IG9	3	1-G	2	0.9648	3.62	0.984
5IG9	3	2-G	1	0.9641	3.64	0.984
5IG9	3	3-G	3	0.9529	3.68	0.984
5IG9	3	4-G	5	0.9507	3.68	0.984
5IG9	3	5-G	4	0.9542	3.68	0.984
5IG9	3	1-H	2	0.9868	0.72	0.981
5IG9	3	2-H	1	0.9872	0.69	0.981
5IG9	3	3-H	3	0.9710	1.12	0.981
5IG9	3	4-H	5	0.9655	1.26	0.981
5IG9	3	5-H	4	0.9729	1.07	0.981
7DRM	3	1-A	5	0.9450	2.52	1.000
7DRM	3	2-A	2	0.9493	2.45	1.000
7DRM	3	3-A	1	0.9475	2.44	1.000
7DRM	3	4-A	3	0.9312	2.68	1.000
7DRM	3	5-A	4	0.9242	2.68	1.000
7DRM	3	1-B	5	0.9553	1.52	1.000
7DRM	3	2-B	2	0.9512	1.60	1.000
7DRM	3	3-B	1	0.9556	1.52	1.000
7DRM	3	4-B	3	0.9463	1.76	1.000
7DRM	3	5-B	4	0.9500	1.66	1.000
7DRM	3	1-C	5	0.9434	2.51	1.000
7DRM	3	2-C	2	0.9479	2.45	1.000
7DRM	3	3-C	1	0.9461	2.43	1.000
7DRM	3	4-C	3	0.9297	2.66	1.000
7DRM	3	5-C	4	0.9220	2.68	1.000
7DRM	3	1-D	5	0.9261	2.91	0.994
7DRM	3	2-D	2	0.9230	2.93	0.994
7DRM	3	3-D	1	0.9260	2.90	0.994
7DRM	3	4-D	3	0.9150	3.06	0.994
7DRM	3	5-D	4	0.9173	2.99	0.994
7M4S	3	1-A	5	0.9175	2.88	1.000
7M4S	3	2-A	2	0.9213	2.81	1.000
7M4S	3	3-A	1	0.9173	2.84	1.000
7M4S	3	4-A	3	0.9113	2.94	1.000
7M4S	3	5-A	4	0.9115	2.99	1.000
7M4S	3	1-B	5	0.9220	2.64	1.000
7M4S	3	2-B	2	0.9254	2.57	1.000
7M4S	3	3-B	1	0.9163	2.65	1.000
7M4S	3	4-B	3	0.9111	2.76	1.000
7M4S	3	5-B	4	0.9107	2.76	1.000
7M4S	3	1-C	5	0.9192	3.57	1.000
7M4S	3	2-C	2	0.9232	3.52	1.000
7M4S	3	3-C	1	0.9218	3.44	1.000
7M4S	3	4-C	3	0.9171	3.54	1.000
7M4S	3	5-C	4	0.9152	3.58	1.000
7M4S	3	1-D	5	0.9311	4.02	1.000
7M4S	3	2-D	2	0.9368	3.97	1.000
7M4S	3	3-D	1	0.9335	3.91	1.000
7M4S	3	4-D	3	0.9273	4.00	1.000
7M4S	3	5-D	4	0.9273	4.03	1.000
7MGV	3	1-A	5	0.9263	4.11	1.000
7MGV	3	2-A	3	0.9192	4.21	1.000
7MGV	3	3-A	1	0.9244	4.09	1.000
7MGV	3	4-A	4	0.9227	4.31	1.000
7MGV	3	5-A	2	0.9242	4.14	1.000
7MGV	3	1-B	5	0.9827	0.91	1.000
7MGV	3	2-B	3	0.9754	1.09	1.000

PDB ID:	Recycle Number:	Model Number and PDB Reference Chain:	Rank:	TMscore:	RMSD(Å):	Sequence Identity:
7MGV	3	3-B	1	0.9809	0.95	1.000
7MGV	3	4-B	4	0.9616	1.58	1.000
7MGV	3	5-B	2	0.9817	0.93	1.000
3VPB	12	1-A	3	0.9906	0.61	1.000
3VPB	12	2-A	5	0.9910	0.60	1.000
3VPB	12	3-A	1	0.9914	0.58	1.000
3VPB	12	4-A	4	0.9900	0.63	1.000
3VPB	12	5-A	2	0.9885	0.68	1.000
3VPB	12	1-B	3	0.9899	0.64	1.000
3VPB	12	2-B	5	0.9904	0.62	1.000
3VPB	12	3-B	1	0.9909	0.60	1.000
3VPB	12	4-B	4	0.9895	0.65	1.000
3VPB	12	5-B	2	0.9879	0.70	1.000
3VPB	12	1-C	3	0.9928	0.53	1.000
3VPB	12	2-C	5	0.9937	0.49	1.000
3VPB	12	3-C	1	0.9934	0.51	1.000
3VPB	12	4-C	4	0.9944	0.47	1.000
3VPB	12	5-C	2	0.9915	0.58	1.000
3VPB	12	1-D	3	0.9930	0.52	1.000
3VPB	12	2-D	5	0.9941	0.48	1.000
3VPB	12	3-D	1	0.9938	0.49	1.000
3VPB	12	4-D	4	0.9945	0.46	1.000
3VPB	12	5-D	2	0.9918	0.57	1.000
3VPD	12	1-A	3	0.9939	0.54	1.000
3VPD	12	2-A	1	0.9929	0.58	1.000
3VPD	12	3-A	2	0.9802	0.93	1.000
3VPD	12	4-A	4	0.9830	0.86	1.000
3VPD	12	5-A	5	0.9807	0.92	1.000
3VPD	12	1-B	3	0.9898	0.65	1.000
3VPD	12	2-B	1	0.9892	0.67	1.000
3VPD	12	3-B	2	0.9878	0.72	1.000
3VPD	12	4-B	4	0.9883	0.70	1.000
3VPD	12	5-B	5	0.9865	0.74	1.000
5IG8	12	1-A	5	0.9195	3.05	1.000
5IG8	12	2-A	4	0.9229	2.82	1.000
5IG8	12	3-A	1	0.8829	3.92	1.000
5IG8	12	4-A	3	0.8814	3.94	1.000
5IG8	12	5-A	2	0.8868	3.73	1.000
5IG8	12	1-B	5	0.9922	0.53	1.000
5IG8	12	2-B	4	0.9917	0.55	1.000
5IG8	12	3-B	1	0.9812	0.81	1.000
5IG8	12	4-B	3	0.9798	0.84	1.000
5IG8	12	5-B	2	0.9804	0.83	1.000
5IG9	12	1-A	2	0.9600	3.87	0.981
5IG9	12	2-A	1	0.9600	3.87	0.981
5IG9	12	3-A	3	0.9430	3.97	0.981
5IG9	12	4-A	5	0.9370	4.03	0.981
5IG9	12	5-A	4	0.9481	3.94	0.981
5IG9	12	1-B	2	0.9904	0.82	0.980
5IG9	12	2-B	1	0.9909	0.81	0.980
5IG9	12	3-B	3	0.9708	1.27	0.980
5IG9	12	4-B	5	0.9640	1.41	0.980
5IG9	12	5-B	4	0.9763	1.14	0.980
5IG9	12	1-C	2	0.9691	3.16	0.984
5IG9	12	2-C	1	0.9692	3.16	0.984
5IG9	12	3-C	3	0.9539	3.27	0.984
5IG9	12	4-C	5	0.9502	3.31	0.984
5IG9	12	5-C	4	0.9576	3.26	0.984
5IG9	12	1-D	2	0.9920	0.80	0.984
5IG9	12	2-D	1	0.9918	0.81	0.984
5IG9	12	3-D	3	0.9755	1.19	0.984
5IG9	12	4-D	5	0.9692	1.31	0.984
5IG9	12	5-D	4	0.9801	1.08	0.984
5IG9	12	1-E	2	0.9650	3.62	0.984

PDB ID:	Recycle Number:	Model Number and PDB Reference Chain:	Rank:	TMscore:	RMSD(Å):	Sequence Identity:
5IG9	12	2-E	1	0.9649	3.61	0.984
5IG9	12	3-E	3	0.9498	3.70	0.984
5IG9	12	4-E	5	0.9463	3.74	0.984
5IG9	12	5-E	4	0.9536	3.68	0.984
5IG9	12	1-F	2	0.9942	0.81	0.983
5IG9	12	2-F	1	0.9942	0.82	0.983
5IG9	12	3-F	3	0.9779	1.19	0.983
5IG9	12	4-F	5	0.9731	1.27	0.983
5IG9	12	5-F	4	0.9820	1.09	0.983
5IG9	12	1-G	2	0.9665	3.61	0.984
5IG9	12	2-G	1	0.9666	3.61	0.984
5IG9	12	3-G	3	0.9518	3.69	0.984
5IG9	12	4-G	5	0.9481	3.73	0.984
5IG9	12	5-G	4	0.9556	3.68	0.984
5IG9	12	1-H	2	0.9889	0.63	0.981
5IG9	12	2-H	1	0.9895	0.60	0.981
5IG9	12	3-H	3	0.9692	1.16	0.981
5IG9	12	4-H	5	0.9626	1.34	0.981
5IG9	12	5-H	4	0.9747	1.02	0.981
7DRM	12	1-A	5	0.9431	2.56	1.000
7DRM	12	2-A	3	0.9475	2.48	1.000
7DRM	12	3-A	1	0.9446	2.49	1.000
7DRM	12	4-A	2	0.9446	2.49	1.000
7DRM	12	5-A	4	0.9165	2.78	1.000
7DRM	12	1-B	5	0.9533	1.56	1.000
7DRM	12	2-B	3	0.9489	1.63	1.000
7DRM	12	3-B	1	0.9564	1.52	1.000
7DRM	12	4-B	2	0.9421	1.87	1.000
7DRM	12	5-B	4	0.9453	1.74	1.000
7DRM	12	1-C	5	0.9415	2.55	1.000
7DRM	12	2-C	3	0.9463	2.48	1.000
7DRM	12	3-C	1	0.9432	2.48	1.000
7DRM	12	4-C	2	0.9250	2.73	1.000
7DRM	12	5-C	4	0.9143	2.77	1.000
7DRM	12	1-D	5	0.9237	2.95	0.994
7DRM	12	2-D	3	0.9208	2.96	0.994
7DRM	12	3-D	1	0.9259	2.92	0.994
7DRM	12	4-D	2	0.9103	3.15	0.994
7DRM	12	5-D	4	0.9121	3.06	0.994
7M4S	12	1-A	4	0.9162	2.92	1.000
7M4S	12	2-A	1	0.9205	2.83	1.000
7M4S	12	3-A	2	0.9183	2.85	1.000
7M4S	12	4-A	3	0.9166	2.84	1.000
7M4S	12	5-A	5	0.9186	2.79	1.000
7M4S	12	1-B	4	0.9206	2.66	1.000
7M4S	12	2-B	1	0.9236	2.60	1.000
7M4S	12	3-B	2	0.9194	2.60	1.000
7M4S	12	4-B	3	0.9160	2.66	1.000
7M4S	12	5-B	5	0.9195	2.56	1.000
7M4S	12	1-C	4	0.9190	3.56	1.000
7M4S	12	2-C	1	0.9226	3.52	1.000
7M4S	12	3-C	2	0.9224	3.46	1.000
7M4S	12	4-C	3	0.9203	3.54	1.000
7M4S	12	5-C	5	0.9194	3.57	1.000
7M4S	12	1-D	4	0.9302	4.02	1.000
7M4S	12	2-D	1	0.9354	3.97	1.000
7M4S	12	3-D	2	0.9333	3.94	1.000
7M4S	12	4-D	3	0.9304	4.01	1.000
7M4S	12	5-D	5	0.9329	4.02	1.000
7MGV	12	1-A	4	0.9248	4.13	1.000
7MGV	12	2-A	5	0.9189	4.23	1.000
7MGV	12	3-A	1	0.9245	4.09	1.000
7MGV	12	4-A	3	0.9242	4.28	1.000
7MGV	12	5-A	2	0.9262	4.12	1.000

PDB ID:	Recycle Number:	Model Number and PDB Reference Chain:	Rank:	TMscore:	RMSD(Å):	Sequence Identity:
7MGV	12	1-B	4	0.9806	0.96	1.000
7MGV	12	2-B	5	0.9747	1.11	1.000
7MGV	12	3-B	1	0.9812	0.95	1.000
7MGV	12	4-B	3	0.9615	1.64	1.000
7MGV	12	5-B	2	0.9837	0.88	1.000
3VPB	24	1-A	3	0.9902	0.62	1.000
3VPB	24	2-A	5	0.9909	0.60	1.000
3VPB	24	3-A	1	0.9913	0.58	1.000
3VPB	24	4-A	4	0.9902	0.62	1.000
3VPB	24	5-A	2	0.9886	0.68	1.000
3VPB	24	1-B	3	0.9896	0.65	1.000
3VPB	24	2-B	5	0.9904	0.62	1.000
3VPB	24	3-B	1	0.9908	0.60	1.000
3VPB	24	4-B	4	0.9897	0.64	1.000
3VPB	24	5-B	2	0.9880	0.70	1.000
3VPB	24	1-C	3	0.9927	0.54	1.000
3VPB	24	2-C	5	0.9938	0.49	1.000
3VPB	24	3-C	1	0.9935	0.50	1.000
3VPB	24	4-C	4	0.9943	0.47	1.000
3VPB	24	5-C	2	0.9917	0.57	1.000
3VPB	24	1-D	3	0.9929	0.53	1.000
3VPB	24	2-D	5	0.9941	0.48	1.000
3VPB	24	3-D	1	0.9938	0.49	1.000
3VPB	24	4-D	4	0.9944	0.47	1.000
3VPB	24	5-D	2	0.9919	0.56	1.000
3VPD	24	1-A	3	0.9939	0.54	1.000
3VPD	24	2-A	1	0.9930	0.58	1.000
3VPD	24	3-A	2	0.9807	0.91	1.000
3VPD	24	4-A	4	0.9829	0.86	1.000
3VPD	24	5-A	5	0.9812	0.90	1.000
3VPD	24	1-B	3	0.9895	0.66	1.000
3VPD	24	2-B	1	0.9893	0.67	1.000
3VPD	24	3-B	2	0.9879	0.71	1.000
3VPD	24	4-B	4	0.9884	0.70	1.000
3VPD	24	5-B	5	0.9867	0.73	1.000
5IG8	24	1-A	5	0.9199	3.00	1.000
5IG8	24	2-A	4	0.9231	2.82	1.000
5IG8	24	3-A	1	0.8829	3.95	1.000
5IG8	24	4-A	3	0.8794	3.98	1.000
5IG8	24	5-A	2	0.8875	3.65	1.000
5IG8	24	1-B	5	0.9922	0.53	1.000
5IG8	24	2-B	4	0.9919	0.54	1.000
5IG8	24	3-B	1	0.9813	0.81	1.000
5IG8	24	4-B	3	0.9788	0.86	1.000
5IG8	24	5-B	2	0.9810	0.82	1.000
5IG9	24	1-A	2	0.9601	3.87	0.981
5IG9	24	2-A	1	0.9601	3.87	0.981
5IG9	24	3-A	4	0.9432	3.96	0.981
5IG9	24	4-A	5	0.9365	4.04	0.981
5IG9	24	5-A	3	0.9469	3.96	0.981
5IG9	24	1-B	2	0.9905	0.82	0.980
5IG9	24	2-B	1	0.9911	0.81	0.980
5IG9	24	3-B	4	0.9713	1.26	0.980
5IG9	24	4-B	5	0.9637	1.42	0.980
5IG9	24	5-B	3	0.9756	1.16	0.980
5IG9	24	1-C	2	0.9692	3.15	0.984
5IG9	24	2-C	1	0.9694	3.16	0.984
5IG9	24	3-C	4	0.9544	3.26	0.984
5IG9	24	4-C	5	0.9498	3.32	0.984
5IG9	24	5-C	3	0.9572	3.26	0.984
5IG9	24	1-D	2	0.9922	0.80	0.984
5IG9	24	2-D	1	0.9919	0.81	0.984
5IG9	24	3-D	4	0.9759	1.18	0.984
5IG9	24	4-D	5	0.9688	1.32	0.984

PDB ID:	Recycle Number:	Model Number and PDB Reference Chain:	Rank:	TMscore:	RMSD(Å):	Sequence Identity:
5IG9	24	5-D	3	0.9795	1.10	0.984
5IG9	24	1-E	2	0.9651	3.61	0.984
5IG9	24	2-E	1	0.9651	3.61	0.984
5IG9	24	3-E	4	0.9503	3.69	0.984
5IG9	24	4-E	5	0.9458	3.74	0.984
5IG9	24	5-E	3	0.9532	3.69	0.984
5IG9	24	1-F	2	0.9943	0.81	0.983
5IG9	24	2-F	1	0.9944	0.81	0.983
5IG9	24	3-F	4	0.9785	1.17	0.983
5IG9	24	4-F	5	0.9728	1.28	0.983
5IG9	24	5-F	3	0.9816	1.10	0.983
5IG9	24	1-G	2	0.9666	3.60	0.984
5IG9	24	2-G	1	0.9669	3.61	0.984
5IG9	24	3-G	4	0.9524	3.69	0.984
5IG9	24	4-G	5	0.9478	3.74	0.984
5IG9	24	5-G	3	0.9553	3.69	0.984
5IG9	24	1-H	2	0.9890	0.63	0.981
5IG9	24	2-H	1	0.9894	0.61	0.981
5IG9	24	3-H	4	0.9697	1.16	0.981
5IG9	24	4-H	5	0.9624	1.34	0.981
5IG9	24	5-H	3	0.9742	1.04	0.981
7DRM	24	1-A	4	0.9434	2.55	1.000
7DRM	24	2-A	2	0.9477	2.48	1.000
7DRM	24	3-A	1	0.9443	2.51	1.000
7DRM	24	4-A	3	0.9239	2.80	1.000
7DRM	24	5-A	5	0.9091	2.87	1.000
7DRM	24	1-B	4	0.9544	1.55	1.000
7DRM	24	2-B	2	0.9503	1.60	1.000
7DRM	24	3-B	1	0.9563	1.52	1.000
7DRM	24	4-B	3	0.9419	1.87	1.000
7DRM	24	5-B	5	0.9412	1.81	1.000
7DRM	24	1-C	4	0.9419	2.55	1.000
7DRM	24	2-C	2	0.9465	2.47	1.000
7DRM	24	3-C	1	0.9428	2.50	1.000
7DRM	24	4-C	3	0.9225	2.77	1.000
7DRM	24	5-C	5	0.9069	2.86	1.000
7DRM	24	1-D	4	0.9247	2.94	0.994
7DRM	24	2-D	2	0.9223	2.94	0.994
7DRM	24	3-D	1	0.9257	2.93	0.994
7DRM	24	4-D	3	0.9099	3.15	0.994
7DRM	24	5-D	5	0.9075	3.11	0.994
7M4S	24	1-A	5	0.9158	2.94	1.000
7M4S	24	2-A	1	0.9196	2.84	1.000
7M4S	24	3-A	2	0.9189	2.89	1.000
7M4S	24	4-A	3	0.9166	2.88	1.000
7M4S	24	5-A	4	0.9179	2.77	1.000
7M4S	24	1-B	5	0.9209	2.66	1.000
7M4S	24	2-B	1	0.9237	2.59	1.000
7M4S	24	3-B	2	0.9205	2.62	1.000
7M4S	24	4-B	3	0.9166	2.67	1.000
7M4S	24	5-B	4	0.9199	2.54	1.000
7M4S	24	1-C	5	0.9178	3.59	1.000
7M4S	24	2-C	1	0.9211	3.54	1.000
7M4S	24	3-C	2	0.9230	3.48	1.000
7M4S	24	4-C	3	0.9200	3.56	1.000
7M4S	24	5-C	4	0.9183	3.57	1.000
7M4S	24	1-D	5	0.9290	4.05	1.000
7M4S	24	2-D	1	0.9338	3.99	1.000
7M4S	24	3-D	2	0.9336	3.97	1.000
7M4S	24	4-D	3	0.9301	4.04	1.000
7M4S	24	5-D	4	0.9318	4.02	1.000
7MGV	24	1-A	5	0.9245	4.14	1.000
7MGV	24	2-A	4	0.9174	4.24	1.000
7MGV	24	3-A	1	0.9251	4.08	1.000

PDB ID:	Recycle Number:	Model Number and PDB Reference Chain:	Rank:	TMscore:	RMSD(Å):	Sequence Identity:
7MGV	24	4-A	3	0.9244	4.29	1.000
7MGV	24	5-A	2	0.9278	4.04	1.000
7MGV	24	1-B	5	0.9806	0.96	1.000
7MGV	24	2-B	4	0.9733	1.14	1.000
7MGV	24	3-B	1	0.9819	0.93	1.000
7MGV	24	4-B	3	0.9619	1.63	1.000
7MGV	24	5-B	2	0.9832	0.89	1.000
3VPB	48	1-A	3	0.9918	0.57	1.000
3VPB	48	2-A	1	0.9919	0.56	1.000
3VPB	48	3-A	2	0.9913	0.59	1.000
3VPB	48	4-A	5	0.9898	0.63	1.000
3VPB	48	5-A	4	0.9897	0.64	1.000
3VPB	48	1-B	3	0.9913	0.59	1.000
3VPB	48	2-B	1	0.9913	0.59	1.000
3VPB	48	3-B	2	0.9907	0.61	1.000
3VPB	48	4-B	5	0.9892	0.66	1.000
3VPB	48	5-B	4	0.9891	0.66	1.000
3VPB	48	1-C	3	0.9975	0.31	1.000
3VPB	48	2-C	1	0.9977	0.30	1.000
3VPB	48	3-C	2	0.9935	0.51	1.000
3VPB	48	4-C	5	0.9944	0.47	1.000
3VPB	48	5-C	4	0.9931	0.52	1.000
3VPB	48	1-D	3	0.9976	0.30	1.000
3VPB	48	2-D	1	0.9978	0.29	1.000
3VPB	48	3-D	2	0.9937	0.50	1.000
3VPB	48	4-D	5	0.9945	0.46	1.000
3VPB	48	5-D	4	0.9933	0.51	1.000
3VPD	48	1-A	2	0.9935	0.56	1.000
3VPD	48	2-A	1	0.9926	0.59	1.000
3VPD	48	3-A	3	0.9797	0.94	1.000
3VPD	48	4-A	4	0.9824	0.88	1.000
3VPD	48	5-A	5	0.9812	0.90	1.000
3VPD	48	1-B	2	0.9877	0.71	1.000
3VPD	48	2-B	1	0.9876	0.72	1.000
3VPD	48	3-B	3	0.9873	0.73	1.000
3VPD	48	4-B	4	0.9880	0.71	1.000
3VPD	48	5-B	5	0.9863	0.74	1.000
5IG8	48	1-A	5	0.9133	3.13	1.000
5IG8	48	2-A	2	0.9162	3.03	1.000
5IG8	48	3-A	1	0.8828	3.99	1.000
5IG8	48	4-A	4	0.8814	3.96	1.000
5IG8	48	5-A	3	0.8853	3.80	1.000
5IG8	48	1-B	5	0.9929	0.50	1.000
5IG8	48	2-B	2	0.9921	0.53	1.000
5IG8	48	3-B	1	0.9818	0.80	1.000
5IG8	48	4-B	4	0.9788	0.86	1.000
5IG8	48	5-B	3	0.9809	0.82	1.000
5IG9	48	1-A	2	0.9618	3.80	0.981
5IG9	48	2-A	1	0.9603	3.81	0.981
5IG9	48	3-A	3	0.9458	3.93	0.981
5IG9	48	4-A	5	0.9383	4.02	0.981
5IG9	48	5-A	4	0.9471	3.97	0.981
5IG9	48	1-B	2	0.9900	0.84	0.980
5IG9	48	2-B	1	0.9896	0.85	0.980
5IG9	48	3-B	3	0.9741	1.19	0.980
5IG9	48	4-B	5	0.9645	1.40	0.980
5IG9	48	5-B	4	0.9752	1.16	0.980
5IG9	48	1-C	2	0.9709	3.11	0.984
5IG9	48	2-C	1	0.9698	3.11	0.984
5IG9	48	3-C	3	0.9562	3.24	0.984
5IG9	48	4-C	5	0.9509	3.31	0.984
5IG9	48	5-C	4	0.9571	3.28	0.984
5IG9	48	1-D	2	0.9914	0.83	0.984
5IG9	48	2-D	1	0.9901	0.87	0.984

PDB ID:	Recycle Number:	Model Number and PDB Reference Chain:	Rank:	TMscore:	RMSD(Å):	Sequence Identity:
5IG9	48	3-D	3	0.9781	1.13	0.984
5IG9	48	4-D	5	0.9698	1.30	0.984
5IG9	48	5-D	4	0.9796	1.08	0.984
5IG9	48	1-E	2	0.9666	3.55	0.984
5IG9	48	2-E	1	0.9653	3.55	0.984
5IG9	48	3-E	3	0.9518	3.67	0.984
5IG9	48	4-E	5	0.9470	3.73	0.984
5IG9	48	5-E	4	0.9530	3.71	0.984
5IG9	48	1-F	2	0.9936	0.83	0.983
5IG9	48	2-F	1	0.9926	0.86	0.983
5IG9	48	3-F	3	0.9803	1.13	0.983
5IG9	48	4-F	5	0.9734	1.27	0.983
5IG9	48	5-F	4	0.9816	1.09	0.983
5IG9	48	1-G	2	0.9682	3.55	0.984
5IG9	48	2-G	1	0.9672	3.55	0.984
5IG9	48	3-G	3	0.9542	3.66	0.984
5IG9	48	4-G	5	0.9488	3.73	0.984
5IG9	48	5-G	4	0.9552	3.70	0.984
5IG9	48	1-H	2	0.9880	0.67	0.981
5IG9	48	2-H	1	0.9876	0.68	0.981
5IG9	48	3-H	3	0.9722	1.09	0.981
5IG9	48	4-H	5	0.9631	1.32	0.981
5IG9	48	5-H	4	0.9743	1.03	0.981
7DRM	48	1-A	2	0.9774	2.09	1.000
7DRM	48	2-A	1	0.9773	2.11	1.000
7DRM	48	3-A	3	0.9449	2.51	1.000
7DRM	48	4-A	4	0.9288	2.63	1.000
7DRM	48	5-A	5	0.9356	2.51	1.000
7DRM	48	1-B	2	0.9589	1.47	1.000
7DRM	48	2-B	1	0.9582	1.48	1.000
7DRM	48	3-B	3	0.9545	1.58	1.000
7DRM	48	4-B	4	0.9466	1.62	1.000
7DRM	48	5-B	5	0.9558	1.46	1.000
7DRM	48	1-C	2	0.9759	2.09	1.000
7DRM	48	2-C	1	0.9760	2.11	1.000
7DRM	48	3-C	3	0.9433	2.49	1.000
7DRM	48	4-C	4	0.9262	2.64	1.000
7DRM	48	5-C	5	0.9325	2.53	1.000
7DRM	48	1-D	2	0.9329	2.84	0.994
7DRM	48	2-D	1	0.9322	2.84	0.994
7DRM	48	3-D	3	0.9234	2.98	0.994
7DRM	48	4-D	4	0.9138	3.02	0.994
7DRM	48	5-D	5	0.9257	2.85	0.994
7M4S	48	1-A	3	0.9340	2.68	1.000
7M4S	48	2-A	1	0.9307	2.70	1.000
7M4S	48	3-A	2	0.9209	2.84	1.000
7M4S	48	4-A	4	0.9204	2.85	1.000
7M4S	48	5-A	5	0.9192	2.71	1.000
7M4S	48	1-B	3	0.9407	2.41	1.000
7M4S	48	2-B	1	0.9373	2.43	1.000
7M4S	48	3-B	2	0.9221	2.58	1.000
7M4S	48	4-B	4	0.9198	2.65	1.000
7M4S	48	5-B	5	0.9209	2.52	1.000
7M4S	48	1-C	3	0.9265	3.54	1.000
7M4S	48	2-C	1	0.9260	3.51	1.000
7M4S	48	3-C	2	0.9241	3.47	1.000
7M4S	48	4-C	4	0.9219	3.56	1.000
7M4S	48	5-C	5	0.9180	3.59	1.000
7M4S	48	1-D	3	0.9415	3.97	1.000
7M4S	48	2-D	1	0.9409	3.93	1.000
7M4S	48	3-D	2	0.9355	3.94	1.000
7M4S	48	4-D	4	0.9340	4.02	1.000
7M4S	48	5-D	5	0.9323	4.02	1.000
7MGV	48	1-A	2	0.9354	4.01	1.000

PDB ID:	Recycle Number:	Model Number and PDB Reference Chain:	Rank:	TMscore:	RMSD(Å):	Sequence Identity:
7MGV	48	2-A	1	0.9331	4.03	1.000
7MGV	48	3-A	3	0.9253	4.06	1.000
7MGV	48	4-A	5	0.9238	4.28	1.000
7MGV	48	5-A	4	0.9288	3.98	1.000
7MGV	48	1-B	2	0.9963	0.42	1.000
7MGV	48	2-B	1	0.9946	0.51	1.000
7MGV	48	3-B	3	0.9816	0.93	1.000
7MGV	48	4-B	5	0.9607	1.64	1.000
7MGV	48	5-B	4	0.9826	0.90	1.000

Table S4. Dimer ATP Grasp Ligase RiPP biosynthetic enzyme US-align results without template or AMBER. Enzymes with multiple available reference subunits are denoted with their corresponding chain letters.

PDB ID:	Recycle Number:	Model Number and PDB Reference Chain:	Rank:	TMscore:	RMSD (Å):	Sequence Identity:
3VPB	3	1-A/B	5	0.9925	0.73	1.000
3VPB	3	2-A/B	4	0.9917	0.77	1.000
3VPB	3	3-A/B	1	0.9927	0.72	1.000
3VPB	3	4-A/B	3	0.9910	0.80	1.000
3VPB	3	5-A/B	2	0.9916	0.78	1.000
3VPB	3	1-C/D	5	0.9958	0.54	1.000
3VPB	3	2-C/D	4	0.9957	0.55	1.000
3VPB	3	3-C/D	1	0.9960	0.53	1.000
3VPB	3	4-C/D	3	0.9949	0.60	1.000
3VPB	3	5-C/D	2	0.9955	0.57	1.000
3VPD	3	1-A/B	3	0.9897	0.87	1.000
3VPD	3	2-A/B	1	0.9907	0.83	1.000
3VPD	3	3-A/B	2	0.9916	0.79	1.000
3VPD	3	4-A/B	4	0.9909	0.82	1.000
3VPD	3	5-A/B	5	0.9907	0.83	1.000
5IG9	3	1-A/B	3	0.9716	3.39	0.981
5IG9	3	2-A/B	1	0.9697	3.55	0.981
5IG9	3	3-A/B	2	0.9693	3.44	0.981
5IG9	3	4-A/B	4	0.9706	3.44	0.981
5IG9	3	5-A/B	5	0.9697	3.52	0.981
5IG9	3	1-C/D	3	0.9773	2.43	0.984
5IG9	3	2-C/D	1	0.9759	2.58	0.984
5IG9	3	3-C/D	2	0.9755	2.47	0.984
5IG9	3	4-C/D	4	0.9765	2.47	0.984
5IG9	3	5-C/D	5	0.9757	2.51	0.984
5IG9	3	1-E/H	3	0.9745	2.69	0.982
5IG9	3	2-E/H	1	0.9732	2.85	0.982
5IG9	3	3-E/H	2	0.9728	2.73	0.982
5IG9	3	4-E/H	4	0.9737	2.74	0.982
5IG9	3	5-E/H	5	0.9725	2.81	0.982
5IG9	3	1-F/G	3	0.9767	2.72	0.984
5IG9	3	2-F/G	1	0.9758	2.88	0.984
5IG9	3	3-F/G	2	0.9751	2.76	0.984
5IG9	3	4-F/G	4	0.9760	2.77	0.984
5IG9	3	5-F/G	5	0.9751	2.84	0.984
7DRM	3	1-A/B	5	0.9748	1.87	1.000
7DRM	3	2-A/B	4	0.9776	1.46	1.000
7DRM	3	3-A/B	2	0.9746	1.91	1.000
7DRM	3	4-A/B	3	0.9786	1.34	1.000
7DRM	3	5-A/B	1	0.9824	1.19	1.000
7DRM	3	1-C/D	5	0.9649	2.46	0.997
7DRM	3	2-C/D	4	0.9682	1.89	0.997
7DRM	3	3-C/D	2	0.9639	2.52	0.997
7DRM	3	4-C/D	3	0.9688	1.84	0.997
7DRM	3	5-C/D	1	0.9745	1.56	0.997
7M4S	3	1-A/D	4	0.9365	3.59	1.000

PDB ID:	Recycle Number:	Model Number and PDB Reference Chain:	Rank:	TMscore:	RMSD (Å):	Sequence Identity:
7M4S	3	2-A/D	2	0.9428	3.41	1.000
7M4S	3	3-A/D	5	0.9447	3.40	1.000
7M4S	3	4-A/D	3	0.9408	3.54	1.000
7M4S	3	5-A/D	1	0.9430	3.50	1.000
7M4S	3	1-B/C	4	0.9336	3.39	1.000
7M4S	3	2-B/C	2	0.9391	3.19	1.000
7M4S	3	3-B/C	5	0.9419	3.18	1.000
7M4S	3	4-B/C	3	0.9386	3.32	1.000
7M4S	3	5-B/C	1	0.9405	3.30	1.000
7MGV	3	1-A/B	1	0.9617	3.01	1.000
7MGV	3	2-A/B	2	0.9624	3.03	1.000
7MGV	3	3-A/B	3	0.9569	3.09	1.000
7MGV	3	4-A/B	4	0.9592	3.08	1.000
7MGV	3	5-A/B	5	0.9594	3.07	1.000
3VPB	12	1-A/B	4	0.9914	0.78	1.000
3VPB	12	2-A/B	5	0.9912	0.79	1.000
3VPB	12	3-A/B	3	0.9915	0.78	1.000
3VPB	12	4-A/B	2	0.9903	0.83	1.000
3VPB	12	5-A/B	1	0.9910	0.80	1.000
3VPB	12	1-C/D	4	0.9951	0.59	1.000
3VPB	12	2-C/D	5	0.9953	0.58	1.000
3VPB	12	3-C/D	3	0.9952	0.58	1.000
3VPB	12	4-C/D	2	0.9945	0.62	1.000
3VPB	12	5-C/D	1	0.9950	0.59	1.000
3VPD	12	1-A/B	5	0.9899	0.87	1.000
3VPD	12	2-A/B	4	0.9901	0.86	1.000
3VPD	12	3-A/B	1	0.9909	0.82	1.000
3VPD	12	4-A/B	2	0.9905	0.84	1.000
3VPD	12	5-A/B	3	0.9897	0.88	1.000
5IG9	12	1-A/B	2	0.9710	3.39	0.981
5IG9	12	2-A/B	1	0.9698	3.42	0.981
5IG9	12	3-A/B	5	0.9688	3.44	0.981
5IG9	12	4-A/B	4	0.9692	3.44	0.981
5IG9	12	5-A/B	3	0.9689	3.45	0.981
5IG9	12	1-C/D	2	0.9768	2.43	0.984
5IG9	12	2-C/D	1	0.9759	2.47	0.984
5IG9	12	3-C/D	5	0.9749	2.48	0.984
5IG9	12	4-C/D	4	0.9751	2.49	0.984
5IG9	12	5-C/D	3	0.9747	2.48	0.984
5IG9	12	1-E/H	2	0.9737	2.69	0.982
5IG9	12	2-E/H	1	0.9733	2.71	0.982
5IG9	12	3-E/H	5	0.9720	2.73	0.982
5IG9	12	4-E/H	4	0.9723	2.74	0.982
5IG9	12	5-E/H	3	0.9717	2.75	0.982
5IG9	12	1-F/G	2	0.9762	2.72	0.984
5IG9	12	2-F/G	1	0.9758	2.74	0.984
5IG9	12	3-F/G	5	0.9743	2.76	0.984
5IG9	12	4-F/G	4	0.9746	2.78	0.984
5IG9	12	5-F/G	3	0.9742	2.78	0.984
7DRM	12	1-A/B	5	0.9777	1.38	1.000
7DRM	12	2-A/B	2	0.9807	1.27	1.000
7DRM	12	3-A/B	3	0.9711	2.00	1.000
7DRM	12	4-A/B	4	0.9780	1.35	1.000
7DRM	12	5-A/B	1	0.9740	1.59	1.000
7DRM	12	1-C/D	5	0.9709	1.64	0.997
7DRM	12	2-C/D	2	0.9706	1.80	0.997
7DRM	12	3-C/D	3	0.9612	2.57	0.997
7DRM	12	4-C/D	4	0.9686	1.81	0.997
7DRM	12	5-C/D	1	0.9667	1.85	0.997
7M4S	12	1-A/D	2	0.9368	3.55	1.000
7M4S	12	2-A/D	1	0.9445	3.37	1.000
7M4S	12	3-A/D	4	0.9472	3.37	1.000
7M4S	12	4-A/D	5	0.9438	3.47	1.000
7M4S	12	5-A/D	4	0.9443	3.47	1.000

PDB ID:	Recycle Number:	Model Number and PDB Reference Chain:	Rank:	TMscore:	RMSD (Å):	Sequence Identity:
7M4S	12	1-B/C	2	0.9324	3.38	1.000
7M4S	12	2-B/C	1	0.9391	3.19	1.000
7M4S	12	3-B/C	4	0.9424	3.18	1.000
7M4S	12	4-B/C	5	0.9399	3.27	1.000
7M4S	12	5-B/C	4	0.9399	3.28	1.000
7MGV	12	1-A/B	5	0.9588	3.02	1.000
7MGV	12	2-A/B	1	0.9584	3.04	1.000
7MGV	12	3-A/B	4	0.9538	3.11	1.000
7MGV	12	4-A/B	3	0.9566	3.10	1.000
7MGV	12	5-A/B	2	0.9582	3.07	1.000
3VPB	24	1-A/B	4	0.9921	0.75	1.000
3VPB	24	2-A/B	1	0.9919	0.76	1.000
3VPB	24	3-A/B	2	0.9917	0.77	1.000
3VPB	24	4-A/B	3	0.9924	0.73	1.000
3VPB	24	5-A/B	5	0.9925	0.73	1.000
3VPB	24	1-C/D	4	0.9944	0.63	1.000
3VPB	24	2-C/D	1	0.9958	0.54	1.000
3VPB	24	3-C/D	2	0.9938	0.66	1.000
3VPB	24	4-C/D	3	0.9953	0.57	1.000
3VPB	24	5-C/D	5	0.9947	0.61	1.000
3VPD	24	1-A/B	5	0.9944	0.65	1.000
3VPD	24	2-A/B	3	0.9947	0.63	1.000
3VPD	24	3-A/B	2	0.9947	0.63	1.000
3VPD	24	4-A/B	1	0.9946	0.64	1.000
3VPD	24	5-A/B	4	0.9946	0.64	1.000
5IG9	24	1-A/B	4	0.9784	3.33	0.981
5IG9	24	2-A/B	2	0.9782	3.35	0.981
5IG9	24	3-A/B	5	0.9782	3.35	0.981
5IG9	24	4-A/B	3	0.9784	3.40	0.981
5IG9	24	5-A/B	1	0.9780	3.36	0.981
5IG9	24	1-C/D	4	0.9839	2.35	0.984
5IG9	24	2-C/D	2	0.9838	2.38	0.984
5IG9	24	3-C/D	5	0.9838	2.37	0.984
5IG9	24	4-C/D	3	0.9840	2.42	0.984
5IG9	24	5-C/D	1	0.9836	2.38	0.984
5IG9	24	1-E/H	4	0.9814	2.61	0.982
5IG9	24	2-E/H	2	0.9813	2.64	0.982
5IG9	24	3-E/H	5	0.9813	2.64	0.982
5IG9	24	4-E/H	3	0.9815	2.69	0.982
5IG9	24	5-E/H	1	0.9811	2.65	0.982
5IG9	24	1-F/G	4	0.9830	2.66	0.984
5IG9	24	2-F/G	2	0.9829	2.69	0.984
5IG9	24	3-F/G	5	0.9829	2.68	0.984
5IG9	24	4-F/G	3	0.9830	2.74	0.984
5IG9	24	5-F/G	1	0.9827	2.70	0.984
7DRM	24	1-A/B	4	0.9758	1.44	1.000
7DRM	24	2-A/B	3	0.9755	1.43	1.000
7DRM	24	3-A/B	1	0.9745	1.50	1.000
7DRM	24	4-A/B	5	0.9779	1.36	1.000
7DRM	24	5-A/B	2	0.9748	1.48	1.000
7DRM	24	1-C/D	4	0.9696	1.67	0.997
7DRM	24	2-C/D	3	0.9671	1.85	0.997
7DRM	24	3-C/D	1	0.9660	1.87	0.997
7DRM	24	4-C/D	5	0.9693	1.77	0.997
7DRM	24	5-C/D	2	0.9688	1.70	0.997
7M4S	24	1-A/D	1	0.9447	3.43	1.000
7M4S	24	2-A/D	5	0.9441	3.32	1.000
7M4S	24	3-A/D	4	0.9499	3.21	1.000
7M4S	24	4-A/D	2	0.9467	3.45	1.000
7M4S	24	5-A/D	3	0.9452	3.33	1.000
7M4S	24	1-B/C	1	0.9401	3.23	1.000
7M4S	24	2-B/C	5	0.9422	3.02	1.000
7M4S	24	3-B/C	4	0.9459	2.99	1.000
7M4S	24	4-B/C	2	0.9430	3.23	1.000

PDB ID:	Recycle Number:	Model Number and PDB Reference Chain:	Rank:	TMscore:	RMSD (Å):	Sequence Identity:
7M4S	24	5-B/C	3	0.9413	3.07	1.000
7MGV	24	1-A/B	5	0.9611	2.95	1.000
7MGV	24	2-A/B	1	0.9592	3.04	1.000
7MGV	24	3-A/B	4	0.9581	3.02	1.000
7MGV	24	4-A/B	3	0.9593	3.09	1.000
7MGV	24	5-A/B	2	0.9602	3.03	1.000
3VPB	48	1-A/B	3	0.9912	0.79	1.000
3VPB	48	2-A/B	4	0.9912	0.79	1.000
3VPB	48	3-A/B	5	0.9911	0.80	1.000
3VPB	48	4-A/B	2	0.9905	0.82	1.000
3VPB	48	5-A/B	1	0.9912	0.79	1.000
3VPB	48	1-C/D	3	0.9951	0.59	1.000
3VPB	48	2-C/D	4	0.9954	0.57	1.000
3VPB	48	3-C/D	5	0.9945	0.62	1.000
3VPB	48	4-C/D	2	0.9948	0.61	1.000
3VPB	48	5-C/D	1	0.9952	0.58	1.000
3VPD	48	1-A/B	5	0.9898	0.87	1.000
3VPD	48	2-A/B	4	0.9903	0.85	1.000
3VPD	48	3-A/B	1	0.9912	0.80	1.000
3VPD	48	4-A/B	3	0.9906	0.83	1.000
3VPD	48	5-A/B	2	0.9897	0.87	1.000
5IG9	48	1-A/B	1	0.9687	3.41	0.981
5IG9	48	2-A/B	2	0.9702	3.41	0.981
5IG9	48	3-A/B	4	0.9692	3.45	0.981
5IG9	48	4-A/B	5	0.9679	3.45	0.981
5IG9	48	5-A/B	3	0.9667	3.48	0.981
5IG9	48	1-C/D	1	0.9746	2.47	0.984
5IG9	48	2-C/D	2	0.9761	2.46	0.984
5IG9	48	3-C/D	4	0.9752	2.49	0.984
5IG9	48	4-C/D	5	0.9739	2.51	0.984
5IG9	48	5-C/D	3	0.9725	2.52	0.984
5IG9	48	1-E/H	1	0.9719	2.72	0.982
5IG9	48	2-E/H	2	0.9734	2.71	0.982
5IG9	48	3-E/H	4	0.9722	2.75	0.982
5IG9	48	4-E/H	5	0.9713	2.76	0.982
5IG9	48	5-E/H	3	0.9695	2.78	0.982
5IG9	48	1-F/G	1	0.9742	2.76	0.984
5IG9	48	2-F/G	2	0.9757	2.74	0.984
5IG9	48	3-F/G	4	0.9747	2.78	0.984
5IG9	48	4-F/G	5	0.9735	2.80	0.984
5IG9	48	5-F/G	3	0.9721	2.81	0.984
7DRM	48	1-A/B	5	0.9776	1.36	1.000
7DRM	48	2-A/B	4	0.9803	1.28	1.000
7DRM	48	3-A/B	2	0.9752	1.44	1.000
7DRM	48	4-A/B	1	0.9758	1.42	1.000
7DRM	48	5-A/B	3	0.9776	1.39	1.000
7DRM	48	1-C/D	5	0.9689	1.79	0.997
7DRM	48	2-C/D	4	0.9702	1.82	0.997
7DRM	48	3-C/D	2	0.9652	1.93	0.997
7DRM	48	4-C/D	1	0.9654	1.91	0.997
7DRM	48	5-C/D	3	0.9705	1.66	0.997
7M4S	48	1-A/D	3	0.9368	3.55	1.000
7M4S	48	2-A/D	1	0.9450	3.35	1.000
7M4S	48	3-A/D	4	0.9473	3.38	1.000
7M4S	48	4-A/D	5	0.9448	3.38	1.000
7M4S	48	5-A/D	2	0.9444	3.42	1.000
7M4S	48	1-B/C	3	0.9308	3.41	1.000
7M4S	48	2-B/C	1	0.9378	3.28	1.000
7M4S	48	3-B/C	4	0.9421	3.21	1.000
7M4S	48	4-B/C	5	0.9398	3.20	1.000
7M4S	48	5-B/C	2	0.9378	3.28	1.000
7MGV	48	1-A/B	5	0.9588	3.01	1.000
7MGV	48	2-A/B	1	0.9583	3.04	1.000
7MGV	48	3-A/B	2	0.9501	3.14	1.000

PDB ID:	Recycle Number:	Model Number and PDB Reference Chain:	Rank:	TMscore:	RMSD (Å):	Sequence Identity:
7MGV	48	4-A/B	3	0.9524	3.16	1.000
7MGV	48	5-A/B	4	0.9584	3.05	1.000
3VPB	72	1-A/B	4	0.9913	0.79	1.000
3VPB	72	2-A/B	3	0.9912	0.79	1.000
3VPB	72	3-A/B	5	0.9908	0.81	1.000
3VPB	72	4-A/B	2	0.9908	0.81	1.000
3VPB	72	5-A/B	1	0.9912	0.79	1.000
3VPB	72	1-C/D	4	0.9951	0.59	1.000
3VPB	72	2-C/D	3	0.9954	0.57	1.000
3VPB	72	3-C/D	5	0.9943	0.64	1.000
3VPB	72	4-C/D	2	0.9949	0.60	1.000
3VPB	72	5-C/D	1	0.9952	0.58	1.000
3VPD	72	1-A/B	5	0.9896	0.88	1.000
3VPD	72	2-A/B	4	0.9902	0.85	1.000
3VPD	72	3-A/B	1	0.9910	0.81	1.000
3VPD	72	4-A/B	2	0.9905	0.84	1.000
3VPD	72	5-A/B	3	0.9896	0.88	1.000
5IG9	72	1-A/B	1	0.9678	3.42	0.981
5IG9	72	2-A/B	2	0.9686	3.42	0.981
5IG9	72	3-A/B	5	0.9693	3.44	0.981
5IG9	72	4-A/B	4	0.9673	3.45	0.981
5IG9	72	5-A/B	3	0.9667	3.46	0.981
5IG9	72	1-C/D	1	0.9738	2.48	0.984
5IG9	72	2-C/D	2	0.9747	2.48	0.984
5IG9	72	3-C/D	5	0.9754	2.49	0.984
5IG9	72	4-C/D	4	0.9733	2.51	0.984
5IG9	72	5-C/D	3	0.9725	2.50	0.984
5IG9	72	1-E/H	1	0.9709	2.72	0.982
5IG9	72	2-E/H	2	0.9720	2.71	0.982
5IG9	72	3-E/H	5	0.9723	2.74	0.982
5IG9	72	4-E/H	4	0.9707	2.76	0.982
5IG9	72	5-E/H	3	0.9695	2.76	0.982
5IG9	72	1-F/G	1	0.9733	2.76	0.984
5IG9	72	2-F/G	2	0.9745	2.75	0.984
5IG9	72	3-F/G	5	0.9748	2.77	0.984
5IG9	72	4-F/G	4	0.9730	2.80	0.984
5IG9	72	5-F/G	3	0.9722	2.79	0.984
7DRM	72	1-A/B	5	0.9774	1.37	1.000
7DRM	72	2-A/B	4	0.9809	1.26	1.000
7DRM	72	3-A/B	2	0.9705	1.71	1.000
7DRM	72	4-A/B	1	0.9760	1.41	1.000
7DRM	72	5-A/B	3	0.9774	1.40	1.000
7DRM	72	1-C/D	5	0.9688	1.79	0.997
7DRM	72	2-C/D	4	0.9706	1.82	0.997
7DRM	72	3-C/D	2	0.9621	2.01	0.997
7DRM	72	4-C/D	1	0.9659	1.86	0.997
7DRM	72	5-C/D	3	0.9703	1.66	0.997
7M4S	72	1-A/D	3	0.9374	3.56	1.000
7M4S	72	2-A/D	1	0.9450	3.36	1.000
7M4S	72	3-A/D	5	0.9473	3.38	1.000
7M4S	72	4-A/D	4	0.9450	3.39	1.000
7M4S	72	5-A/D	2	0.9448	3.39	1.000
7M4S	72	1-B/C	3	0.9311	3.42	1.000
7M4S	72	2-B/C	1	0.9387	3.19	1.000
7M4S	72	3-B/C	5	0.9422	3.21	1.000
7M4S	72	4-B/C	4	0.9402	3.20	1.000
7M4S	72	5-B/C	2	0.9381	3.25	1.000
7MGV	72	1-A/B	5	0.9602	2.99	1.000
7MGV	72	2-A/B	1	0.9595	3.02	1.000
7MGV	72	3-A/B	4	0.9508	3.13	1.000
7MGV	72	4-A/B	2	0.9529	3.14	1.000
7MGV	72	5-A/B	3	0.9596	3.03	1.000

Table S5. Dimer ATP Grasp Ligase RiPP biosynthetic enzyme US-align results with template. Enzymes with multiple available reference subunits are denoted with their corresponding chain letters.

PDB ID:	Recycle Number:	Model Number and PDB Reference Chain:	Rank:	TMscore:	RMSD (Å):	Sequence Identity:
3VPB	3	1-A/B	4	0.9929	0.71	1.000
3VPB	3	2-A/B	2	0.9922	0.75	1.000
3VPB	3	3-A/B	5	0.9925	0.73	1.000
3VPB	3	4-A/B	1	0.9920	0.75	1.000
3VPB	3	5-A/B	3	0.9923	0.74	1.000
3VPB	3	1-C/D	4	0.9953	0.58	1.000
3VPB	3	2-C/D	2	0.9956	0.56	1.000
3VPB	3	3-C/D	5	0.9948	0.60	1.000
3VPB	3	4-C/D	1	0.9952	0.58	1.000
3VPB	3	5-C/D	3	0.9951	0.59	1.000
3VPD	3	1-A/B	5	0.9944	0.65	1.000
3VPD	3	2-A/B	4	0.9952	0.60	1.000
3VPD	3	3-A/B	3	0.9947	0.63	1.000
3VPD	3	4-A/B	1	0.9947	0.63	1.000
3VPD	3	5-A/B	2	0.9947	0.63	1.000
5IG9	3	1-A/B	3	0.9783	3.35	0.981
5IG9	3	2-A/B	2	0.9788	3.40	0.981
5IG9	3	3-A/B	5	0.9779	3.38	0.981
5IG9	3	4-A/B	4	0.9786	3.41	0.981
5IG9	3	5-A/B	1	0.9780	3.39	0.981
5IG9	3	1-C/D	3	0.9839	2.37	0.984
5IG9	3	2-C/D	2	0.9844	2.39	0.984
5IG9	3	3-C/D	5	0.9836	2.40	0.984
5IG9	3	4-C/D	4	0.9842	2.40	0.984
5IG9	3	5-C/D	1	0.9837	2.41	0.984
5IG9	3	1-E/H	3	0.9814	2.63	0.982
5IG9	3	2-E/H	2	0.9818	2.68	0.982
5IG9	3	3-E/H	5	0.9811	2.66	0.982
5IG9	3	4-E/H	4	0.9816	2.69	0.982
5IG9	3	5-E/H	1	0.9812	2.68	0.982
5IG9	3	1-F/G	3	0.9830	2.68	0.984
5IG9	3	2-F/G	2	0.9835	2.73	0.984
5IG9	3	3-F/G	5	0.9827	2.71	0.984
5IG9	3	4-F/G	4	0.9833	2.74	0.984
5IG9	3	5-F/G	1	0.9828	2.73	0.984
7DRM	3	1-A/B	3	0.9711	1.98	1.000
7DRM	3	2-A/B	1	0.9688	2.04	1.000
7DRM	3	3-A/B	4	0.9743	1.92	1.000
7DRM	3	4-A/B	5	0.9766	1.40	1.000
7DRM	3	5-A/B	2	0.9746	1.88	1.000
7DRM	3	1-C/D	3	0.9600	2.65	0.997
7DRM	3	2-C/D	1	0.9593	2.58	0.997
7DRM	3	3-C/D	4	0.9638	2.53	0.997
7DRM	3	4-C/D	5	0.9680	1.82	0.997
7DRM	3	5-C/D	2	0.9647	2.48	0.997
7M4S	3	1-A/D	1	0.9432	3.45	1.000
7M4S	3	2-A/D	4	0.9446	3.29	1.000
7M4S	3	3-A/D	5	0.9477	3.23	1.000
7M4S	3	4-A/D	2	0.9428	3.51	1.000
7M4S	3	5-A/D	3	0.9452	3.34	1.000
7M4S	3	1-B/C	1	0.9402	3.21	1.000
7M4S	3	2-B/C	4	0.9428	3.01	1.000
7M4S	3	3-B/C	5	0.9455	2.97	1.000
7M4S	3	4-B/C	2	0.9408	3.27	1.000
7M4S	3	5-B/C	3	0.9414	3.08	1.000
7MGV	3	1-A/B	3	0.9618	3.02	1.000
7MGV	3	2-A/B	1	0.9626	3.04	1.000

PDB ID:	Recycle Number:	Model Number and PDB Reference Chain:	Rank:	TMscore:	RMSD (Å):	Sequence Identity:
7MGV	3	3-A/B	5	0.9564	3.10	1.000
7MGV	3	4-A/B	4	0.9566	3.14	1.000
7MGV	3	5-A/B	2	0.9604	3.08	1.000
3VPB	12	1-A/B	5	0.9919	0.76	1.000
3VPB	12	2-A/B	3	0.9919	0.76	1.000
3VPB	12	3-A/B	2	0.9917	0.77	1.000
3VPB	12	4-A/B	1	0.9920	0.75	1.000
3VPB	12	5-A/B	4	0.9922	0.74	1.000
3VPB	12	1-C/D	5	0.9945	0.63	1.000
3VPB	12	2-C/D	3	0.9956	0.56	1.000
3VPB	12	3-C/D	2	0.9940	0.65	1.000
3VPB	12	4-C/D	1	0.9950	0.59	1.000
3VPB	12	5-C/D	4	0.9946	0.62	1.000
3VPD	12	1-A/B	5	0.9944	0.65	1.000
3VPD	12	2-A/B	4	0.9947	0.63	1.000
3VPD	12	3-A/B	2	0.9946	0.63	1.000
3VPD	12	4-A/B	1	0.9946	0.63	1.000
3VPD	12	5-A/B	3	0.9946	0.64	1.000
5IG9	12	1-A/B	4	0.9784	3.33	0.981
5IG9	12	2-A/B	1	0.9783	3.38	0.981
5IG9	12	3-A/B	5	0.9780	3.36	0.981
5IG9	12	4-A/B	3	0.9786	3.40	0.981
5IG9	12	5-A/B	2	0.9779	3.37	0.981
5IG9	12	1-C/D	4	0.9839	2.35	0.984
5IG9	12	2-C/D	1	0.9839	2.40	0.984
5IG9	12	3-C/D	5	0.9836	2.38	0.984
5IG9	12	4-C/D	3	0.9842	2.40	0.984
5IG9	12	5-C/D	2	0.9835	2.39	0.984
5IG9	12	1-E/H	4	0.9814	2.61	0.982
5IG9	12	2-E/H	1	0.9815	2.66	0.982
5IG9	12	3-E/H	5	0.9812	2.64	0.982
5IG9	12	4-E/H	3	0.9815	2.68	0.982
5IG9	12	5-E/H	2	0.9810	2.65	0.982
5IG9	12	1-F/G	4	0.9830	2.66	0.984
5IG9	12	2-F/G	1	0.9830	2.71	0.984
5IG9	12	3-F/G	5	0.9828	2.69	0.984
5IG9	12	4-F/G	3	0.9833	2.72	0.984
5IG9	12	5-F/G	2	0.9827	2.70	0.984
7DRM	12	1-A/B	5	0.9691	2.00	1.000
7DRM	12	2-A/B	1	0.9755	1.43	1.000
7DRM	12	3-A/B	2	0.9709	1.99	1.000
7DRM	12	4-A/B	4	0.9773	1.38	1.000
7DRM	12	5-A/B	3	0.9760	1.43	1.000
7DRM	12	1-C/D	5	0.9604	2.53	0.997
7DRM	12	2-C/D	1	0.9670	1.86	0.997
7DRM	12	3-C/D	2	0.9606	2.58	0.997
7DRM	12	4-C/D	4	0.9688	1.78	0.997
7DRM	12	5-C/D	3	0.9699	1.67	0.997
7M4S	12	1-A/D	1	0.9442	3.44	1.000
7M4S	12	2-A/D	2	0.9440	3.32	1.000
7M4S	12	3-A/D	4	0.9489	3.22	1.000
7M4S	12	4-A/D	3	0.9457	3.45	1.000
7M4S	12	5-A/D	5	0.9448	3.33	1.000
7M4S	12	1-B/C	1	0.9401	3.23	1.000
7M4S	12	2-B/C	2	0.9421	3.02	1.000
7M4S	12	3-B/C	4	0.9456	2.98	1.000
7M4S	12	4-B/C	3	0.9425	3.22	1.000
7M4S	12	5-B/C	5	0.9411	3.07	1.000
7MGV	12	1-A/B	4	0.9607	2.97	1.000
7MGV	12	2-A/B	1	0.9595	3.05	1.000
7MGV	12	3-A/B	5	0.9571	3.04	1.000
7MGV	12	4-A/B	3	0.9586	3.10	1.000
7MGV	12	5-A/B	2	0.9603	3.04	1.000
3VPB	24	1-A/B	4	0.9921	0.75	1.000

PDB ID:	Recycle Number:	Model Number and PDB Reference Chain:	Rank:	TMscore:	RMSD (Å):	Sequence Identity:
3VPB	24	2-A/B	1	0.9919	0.76	1.000
3VPB	24	3-A/B	2	0.9917	0.77	1.000
3VPB	24	4-A/B	3	0.9924	0.73	1.000
3VPB	24	5-A/B	5	0.9925	0.73	1.000
3VPB	24	1-C/D	4	0.9944	0.63	1.000
3VPB	24	2-C/D	1	0.9958	0.54	1.000
3VPB	24	3-C/D	2	0.9938	0.66	1.000
3VPB	24	4-C/D	3	0.9953	0.57	1.000
3VPB	24	5-C/D	5	0.9947	0.61	1.000
3VPD	24	1-A/B	5	0.9944	0.65	1.000
3VPD	24	2-A/B	3	0.9947	0.63	1.000
3VPD	24	3-A/B	2	0.9947	0.63	1.000
3VPD	24	4-A/B	1	0.9946	0.64	1.000
3VPD	24	5-A/B	4	0.9946	0.64	1.000
5IG9	24	1-A/B	4	0.9784	3.33	0.981
5IG9	24	2-A/B	2	0.9782	3.35	0.981
5IG9	24	3-A/B	5	0.9782	3.35	0.981
5IG9	24	4-A/B	3	0.9784	3.40	0.981
5IG9	24	5-A/B	1	0.9780	3.36	0.981
5IG9	24	1-C/D	4	0.9839	2.35	0.984
5IG9	24	2-C/D	2	0.9838	2.38	0.984
5IG9	24	3-C/D	5	0.9838	2.37	0.984
5IG9	24	4-C/D	3	0.9840	2.42	0.984
5IG9	24	5-C/D	1	0.9836	2.38	0.984
5IG9	24	1-E/H	4	0.9814	2.61	0.982
5IG9	24	2-E/H	2	0.9813	2.64	0.982
5IG9	24	3-E/H	5	0.9813	2.64	0.982
5IG9	24	4-E/H	3	0.9815	2.69	0.982
5IG9	24	5-E/H	1	0.9811	2.65	0.982
5IG9	24	1-F/G	4	0.9830	2.66	0.984
5IG9	24	2-F/G	2	0.9829	2.69	0.984
5IG9	24	3-F/G	5	0.9829	2.68	0.984
5IG9	24	4-F/G	3	0.9830	2.74	0.984
5IG9	24	5-F/G	1	0.9827	2.70	0.984
7DRM	24	1-A/B	4	0.9758	1.44	1.000
7DRM	24	2-A/B	3	0.9755	1.43	1.000
7DRM	24	3-A/B	1	0.9745	1.50	1.000
7DRM	24	4-A/B	5	0.9779	1.36	1.000
7DRM	24	5-A/B	2	0.9748	1.48	1.000
7DRM	24	1-C/D	4	0.9696	1.67	0.997
7DRM	24	2-C/D	3	0.9671	1.85	0.997
7DRM	24	3-C/D	1	0.9660	1.87	0.997
7DRM	24	4-C/D	5	0.9693	1.77	0.997
7DRM	24	5-C/D	2	0.9688	1.70	0.997
7M4S	24	1-A/D	1	0.9447	3.43	1.000
7M4S	24	2-A/D	5	0.9441	3.32	1.000
7M4S	24	3-A/D	4	0.9499	3.21	1.000
7M4S	24	4-A/D	2	0.9467	3.45	1.000
7M4S	24	5-A/D	3	0.9452	3.33	1.000
7M4S	24	1-B/C	1	0.9401	3.23	1.000
7M4S	24	2-B/C	5	0.9422	3.02	1.000
7M4S	24	3-B/C	4	0.9459	2.99	1.000
7M4S	24	4-B/C	2	0.9430	3.23	1.000
7M4S	24	5-B/C	3	0.9413	3.07	1.000
7MGV	24	1-A/B	5	0.9611	2.95	1.000
7MGV	24	2-A/B	1	0.9592	3.04	1.000
7MGV	24	3-A/B	4	0.9581	3.02	1.000
7MGV	24	4-A/B	3	0.9593	3.09	1.000
7MGV	24	5-A/B	2	0.9602	3.03	1.000
3VPB	48	1-A/B	5	0.9921	0.75	1.000
3VPB	48	2-A/B	3	0.9919	0.76	1.000
3VPB	48	3-A/B	1	0.9917	0.77	1.000
3VPB	48	4-A/B	2	0.9925	0.73	1.000
3VPB	48	5-A/B	4	0.9926	0.73	1.000

PDB ID:	Recycle Number:	Model Number and PDB Reference Chain:	Rank:	TMscore:	RMSD (Å):	Sequence Identity:
3VPB	48	1-C/D	5	0.9943	0.64	1.000
3VPB	48	2-C/D	3	0.9958	0.55	1.000
3VPB	48	3-C/D	1	0.9937	0.67	1.000
3VPB	48	4-C/D	2	0.9953	0.58	1.000
3VPB	48	5-C/D	4	0.9947	0.61	1.000
3VPD	48	1-A/B	5	0.9944	0.65	1.000
3VPD	48	2-A/B	4	0.9947	0.63	1.000
3VPD	48	3-A/B	3	0.9947	0.63	1.000
3VPD	48	4-A/B	1	0.9946	0.64	1.000
3VPD	48	5-A/B	2	0.9946	0.64	1.000
5IG9	48	1-A/B	3	0.9784	3.33	0.981
5IG9	48	2-A/B	1	0.9782	3.36	0.981
5IG9	48	3-A/B	5	0.9781	3.36	0.981
5IG9	48	4-A/B	4	0.9781	3.37	0.981
5IG9	48	5-A/B	2	0.9780	3.37	0.981
5IG9	48	1-C/D	3	0.9839	2.35	0.984
5IG9	48	2-C/D	1	0.9838	2.38	0.984
5IG9	48	3-C/D	5	0.9837	2.38	0.984
5IG9	48	4-C/D	4	0.9837	2.40	0.984
5IG9	48	5-C/D	2	0.9836	2.39	0.984
5IG9	48	1-E/H	3	0.9815	2.61	0.982
5IG9	48	2-E/H	1	0.9814	2.64	0.982
5IG9	48	3-E/H	5	0.9813	2.64	0.982
5IG9	48	4-E/H	4	0.9813	2.66	0.982
5IG9	48	5-E/H	2	0.9811	2.66	0.982
5IG9	48	1-F/G	3	0.9831	2.66	0.984
5IG9	48	2-F/G	1	0.9829	2.69	0.984
5IG9	48	3-F/G	5	0.9828	2.69	0.984
5IG9	48	4-F/G	4	0.9828	2.71	0.984
5IG9	48	5-F/G	2	0.9827	2.71	0.984
7DRM	48	1-A/B	4	0.9754	1.45	1.000
7DRM	48	2-A/B	3	0.9744	1.46	1.000
7DRM	48	3-A/B	1	0.9750	1.47	1.000
7DRM	48	4-A/B	5	0.9778	1.36	1.000
7DRM	48	5-A/B	2	0.9748	1.47	1.000
7DRM	48	1-C/D	4	0.9691	1.69	0.997
7DRM	48	2-C/D	3	0.9661	1.89	0.997
7DRM	48	3-C/D	1	0.9666	1.80	0.997
7DRM	48	4-C/D	5	0.9693	1.77	0.997
7DRM	48	5-C/D	2	0.9687	1.71	0.997
7M4S	48	1-A/D	1	0.9437	3.43	1.000
7M4S	48	2-A/D	3	0.9432	3.32	1.000
7M4S	48	3-A/D	5	0.9490	3.21	1.000
7M4S	48	4-A/D	2	0.9462	3.45	1.000
7M4S	48	5-A/D	4	0.9441	3.34	1.000
7M4S	48	1-B/C	1	0.9395	3.22	1.000
7M4S	48	2-B/C	3	0.9416	3.02	1.000
7M4S	48	3-B/C	5	0.9453	2.98	1.000
7M4S	48	4-B/C	2	0.9422	3.24	1.000
7M4S	48	5-B/C	4	0.9405	3.07	1.000
7MGV	48	1-A/B	3	0.9597	2.97	1.000
7MGV	48	2-A/B	1	0.9581	3.06	1.000
7MGV	48	3-A/B	5	0.9567	3.04	1.000
7MGV	48	4-A/B	4	0.9579	3.12	1.000
7MGV	48	5-A/B	2	0.9572	3.07	1.000
3VPB	72	1-A/B	5	0.9920	0.75	1.000
3VPB	72	2-A/B	3	0.9918	0.76	1.000
3VPB	72	3-A/B	2	0.9916	0.77	1.000
3VPB	72	4-A/B	1	0.9926	0.73	1.000
3VPB	72	5-A/B	4	0.9924	0.74	1.000
3VPB	72	1-C/D	5	0.9943	0.64	1.000
3VPB	72	2-C/D	3	0.9957	0.55	1.000
3VPB	72	3-C/D	2	0.9937	0.67	1.000
3VPB	72	4-C/D	1	0.9954	0.57	1.000

PDB ID:	Recycle Number:	Model Number and PDB Reference Chain:	Rank:	TMscore:	RMSD (Å):	Sequence Identity:
3VPB	72	5-C/D	4	0.9946	0.62	1.000
3VPD	72	1-A/B	5	0.9945	0.65	1.000
3VPD	72	2-A/B	4	0.9948	0.62	1.000
3VPD	72	3-A/B	2	0.9948	0.63	1.000
3VPD	72	4-A/B	1	0.9947	0.63	1.000
3VPD	72	5-A/B	3	0.9947	0.63	1.000
5IG9	72	1-A/B	4	0.9784	3.33	0.981
5IG9	72	2-A/B	2	0.9781	3.35	0.981
5IG9	72	3-A/B	5	0.9780	3.36	0.981
5IG9	72	4-A/B	3	0.9781	3.36	0.981
5IG9	72	5-A/B	1	0.9779	3.37	0.981
5IG9	72	1-C/D	4	0.9839	2.35	0.984
5IG9	72	2-C/D	2	0.9837	2.38	0.984
5IG9	72	3-C/D	5	0.9837	2.38	0.984
5IG9	72	4-C/D	3	0.9837	2.39	0.984
5IG9	72	5-C/D	1	0.9835	2.39	0.984
5IG9	72	1-E/H	4	0.9814	2.61	0.982
5IG9	72	2-E/H	2	0.9812	2.64	0.982
5IG9	72	3-E/H	5	0.9812	2.64	0.982
5IG9	72	4-E/H	3	0.9812	2.65	0.982
5IG9	72	5-E/H	1	0.9809	2.65	0.982
5IG9	72	1-F/G	4	0.9830	2.66	0.984
5IG9	72	2-F/G	2	0.9828	2.69	0.984
5IG9	72	3-F/G	5	0.9828	2.69	0.984
5IG9	72	4-F/G	3	0.9828	2.70	0.984
5IG9	72	5-F/G	1	0.9826	2.70	0.984
7DRM	72	1-A/B	4	0.9750	1.47	1.000
7DRM	72	2-A/B	3	0.9751	1.44	1.000
7DRM	72	3-A/B	1	0.9751	1.46	1.000
7DRM	72	4-A/B	5	0.9797	1.30	1.000
7DRM	72	5-A/B	2	0.9748	1.47	1.000
7DRM	72	1-C/D	4	0.9686	1.71	0.997
7DRM	72	2-C/D	3	0.9667	1.87	0.997
7DRM	72	3-C/D	1	0.9667	1.80	0.997
7DRM	72	4-C/D	5	0.9709	1.72	0.997
7DRM	72	5-C/D	2	0.9688	1.70	0.997
7M4S	72	1-A/D	2	0.9441	3.43	1.000
7M4S	72	2-A/D	4	0.9439	3.32	1.000
7M4S	72	3-A/D	5	0.9497	3.21	1.000
7M4S	72	4-A/D	1	0.9468	3.46	1.000
7M4S	72	5-A/D	3	0.9479	3.25	1.000
7M4S	72	1-B/C	2	0.9396	3.22	1.000
7M4S	72	2-B/C	4	0.9419	3.02	1.000
7M4S	72	3-B/C	5	0.9458	2.98	1.000
7M4S	72	4-B/C	1	0.9427	3.25	1.000
7M4S	72	5-B/C	3	0.9431	3.03	1.000
7MGV	72	1-A/B	5	0.9609	2.95	1.000
7MGV	72	2-A/B	1	0.9591	3.05	1.000
7MGV	72	3-A/B	3	0.9581	3.02	1.000
7MGV	72	4-A/B	4	0.9583	3.10	1.000
7MGV	72	5-A/B	2	0.9582	3.05	1.000

Table S6. Dimer ATP Grasp Ligase RiPP biosynthetic enzyme US-align results with template and AMBER. Enzymes with multiple available reference subunits are denoted with their corresponding chain letters.

PDB ID:	Recycle Number:	Model Number and PDB Reference Chain:	Rank:	TMscore:	RMSD (Å):	Sequence Identity:
3VPB	3	1-A/B	4	0.9929	0.71	1.000
3VPB	3	2-A/B	2	0.9922	0.75	1.000
3VPB	3	3-A/B	5	0.9922	0.75	1.000
3VPB	3	4-A/B	1	0.9920	0.76	1.000

PDB ID:	Recycle Number:	Model Number and PDB Reference Chain:	Rank:	TMscore:	RMSD (Å):	Sequence Identity:
3VPB	3	5-A/B	3	0.9925	0.73	1.000
3VPB	3	1-C/D	4	0.9952	0.58	1.000
3VPB	3	2-C/D	2	0.9955	0.56	1.000
3VPB	3	3-C/D	5	0.9948	0.61	1.000
3VPB	3	4-C/D	1	0.9951	0.59	1.000
3VPB	3	5-C/D	3	0.9950	0.59	1.000
3VPD	3	1-A/B	5	0.9943	0.65	1.000
3VPD	3	2-A/B	4	0.9951	0.61	1.000
3VPD	3	3-A/B	3	0.9946	0.63	1.000
3VPD	3	4-A/B	1	0.9946	0.64	1.000
3VPD	3	5-A/B	2	0.9946	0.64	1.000
5IG9	3	1-A/B	3	0.9782	3.35	0.981
5IG9	3	2-A/B	2	0.9786	3.41	0.981
5IG9	3	3-A/B	5	0.9779	3.38	0.981
5IG9	3	4-A/B	4	0.9785	3.41	0.981
5IG9	3	5-A/B	1	0.9780	3.39	0.981
5IG9	3	1-C/D	3	0.9837	2.37	0.984
5IG9	3	2-C/D	2	0.9842	2.40	0.984
5IG9	3	3-C/D	5	0.9835	2.40	0.984
5IG9	3	4-C/D	4	0.9841	2.41	0.984
5IG9	3	5-C/D	1	0.9836	2.41	0.984
5IG9	3	1-E/H	3	0.9812	2.63	0.982
5IG9	3	2-E/H	2	0.9816	2.69	0.982
5IG9	3	3-E/H	5	0.9811	2.67	0.982
5IG9	3	4-E/H	4	0.9814	2.69	0.982
5IG9	3	5-E/H	1	0.9811	2.68	0.982
5IG9	3	1-F/G	3	0.9828	2.69	0.984
5IG9	3	2-F/G	2	0.9833	2.74	0.984
5IG9	3	3-F/G	5	0.9826	2.72	0.984
5IG9	3	4-F/G	4	0.9832	2.74	0.984
5IG9	3	5-F/G	1	0.9827	2.73	0.984
7DRM	3	1-A/B	3	0.9709	1.99	1.000
7DRM	3	2-A/B	1	0.9684	2.04	1.000
7DRM	3	3-A/B	4	0.9741	1.93	1.000
7DRM	3	4-A/B	5	0.9764	1.41	1.000
7DRM	3	5-A/B	2	0.9744	1.88	1.000
7DRM	3	1-C/D	3	0.9598	2.65	0.997
7DRM	3	2-C/D	1	0.9590	2.59	0.997
7DRM	3	3-C/D	4	0.9635	2.53	0.997
7DRM	3	4-C/D	5	0.9678	1.83	0.997
7DRM	3	5-C/D	2	0.9645	2.48	0.997
7M4S	3	1-A/D	1	0.9430	3.46	1.000
7M4S	3	2-A/D	4	0.9442	3.31	1.000
7M4S	3	3-A/D	5	0.9473	3.24	1.000
7M4S	3	4-A/D	2	0.9426	3.52	1.000
7M4S	3	5-A/D	3	0.9450	3.35	1.000
7M4S	3	1-B/C	1	0.9399	3.23	1.000
7M4S	3	2-B/C	4	0.9424	3.02	1.000
7M4S	3	3-B/C	5	0.9451	2.98	1.000
7M4S	3	4-B/C	2	0.9406	3.28	1.000
7M4S	3	5-B/C	3	0.9412	3.08	1.000
7MGV	3	1-A/B	3	0.9617	3.03	1.000
7MGV	3	2-A/B	1	0.9624	3.05	1.000
7MGV	3	3-A/B	5	0.9562	3.10	1.000
7MGV	3	4-A/B	4	0.9565	3.15	1.000
7MGV	3	5-A/B	2	0.9602	3.08	1.000
3VPB	12	1-A/B	5	0.9919	0.76	1.000
3VPB	12	2-A/B	3	0.9919	0.76	1.000
3VPB	12	3-A/B	2	0.9916	0.77	1.000
3VPB	12	4-A/B	1	0.9920	0.76	1.000
3VPB	12	5-A/B	4	0.9922	0.75	1.000
3VPB	12	1-C/D	5	0.9944	0.63	1.000
3VPB	12	2-C/D	3	0.9955	0.56	1.000
3VPB	12	3-C/D	2	0.9939	0.66	1.000

PDB ID:	Recycle Number:	Model Number and PDB Reference Chain:	Rank:	TMscore:	RMSD (Å):	Sequence Identity:
3VPB	12	4-C/D	1	0.9949	0.60	1.000
3VPB	12	5-C/D	4	0.9945	0.62	1.000
3VPD	12	1-A/B	5	0.9943	0.66	1.000
3VPD	12	2-A/B	4	0.9946	0.64	1.000
3VPD	12	3-A/B	2	0.9946	0.64	1.000
3VPD	12	4-A/B	1	0.9946	0.64	1.000
3VPD	12	5-A/B	3	0.9945	0.64	1.000
5IG9	12	1-A/B	4	0.9782	3.33	0.981
5IG9	12	2-A/B	1	0.9781	3.38	0.981
5IG9	12	3-A/B	5	0.9779	3.36	0.981
5IG9	12	4-A/B	3	0.9785	3.39	0.981
5IG9	12	5-A/B	2	0.9779	3.36	0.981
5IG9	12	1-C/D	4	0.9838	2.35	0.984
5IG9	12	2-C/D	1	0.9837	2.41	0.984
5IG9	12	3-C/D	5	0.9836	2.39	0.984
5IG9	12	4-C/D	3	0.9841	2.40	0.984
5IG9	12	5-C/D	2	0.9835	2.39	0.984
5IG9	12	1-E/H	4	0.9812	2.61	0.982
5IG9	12	2-E/H	1	0.9812	2.67	0.982
5IG9	12	3-E/H	5	0.9811	2.65	0.982
5IG9	12	4-E/H	3	0.9814	2.68	0.982
5IG9	12	5-E/H	2	0.9809	2.65	0.982
5IG9	12	1-F/G	4	0.9829	2.66	0.984
5IG9	12	2-F/G	1	0.9828	2.72	0.984
5IG9	12	3-F/G	5	0.9827	2.70	0.984
5IG9	12	4-F/G	3	0.9832	2.73	0.984
5IG9	12	5-F/G	2	0.9826	2.70	0.984
7DRM	12	1-A/B	5	0.9688	2.01	1.000
7DRM	12	2-A/B	1	0.9752	1.44	1.000
7DRM	12	3-A/B	2	0.9707	2.00	1.000
7DRM	12	4-A/B	4	0.9771	1.38	1.000
7DRM	12	5-A/B	3	0.9758	1.44	1.000
7DRM	12	1-C/D	5	0.9600	2.54	0.997
7DRM	12	2-C/D	1	0.9667	1.88	0.997
7DRM	12	3-C/D	2	0.9603	2.59	0.997
7DRM	12	4-C/D	4	0.9686	1.79	0.997
7DRM	12	5-C/D	3	0.9697	1.68	0.997
7M4S	12	1-A/D	1	0.9440	3.45	1.000
7M4S	12	2-A/D	2	0.9437	3.34	1.000
7M4S	12	3-A/D	4	0.9485	3.23	1.000
7M4S	12	4-A/D	3	0.9455	3.47	1.000
7M4S	12	5-A/D	5	0.9446	3.34	1.000
7M4S	12	1-B/C	1	0.9399	3.24	1.000
7M4S	12	2-B/C	2	0.9417	3.03	1.000
7M4S	12	3-B/C	4	0.9452	2.99	1.000
7M4S	12	4-B/C	3	0.9423	3.23	1.000
7M4S	12	5-B/C	5	0.9409	3.07	1.000
7MGV	12	1-A/B	4	0.9605	2.98	1.000
7MGV	12	2-A/B	1	0.9593	3.06	1.000
7MGV	12	3-A/B	5	0.9569	3.05	1.000
7MGV	12	4-A/B	3	0.9585	3.11	1.000
7MGV	12	5-A/B	2	0.9602	3.04	1.000
3VPB	24	1-A/B	4	0.9920	0.75	1.000
3VPB	24	2-A/B	1	0.9919	0.76	1.000
3VPB	24	3-A/B	2	0.9917	0.77	1.000
3VPB	24	4-A/B	3	0.9924	0.74	1.000
3VPB	24	5-A/B	5	0.9925	0.73	1.000
3VPB	24	1-C/D	4	0.9943	0.63	1.000
3VPB	24	2-C/D	1	0.9958	0.55	1.000
3VPB	24	3-C/D	2	0.9938	0.67	1.000
3VPB	24	4-C/D	3	0.9952	0.58	1.000
3VPB	24	5-C/D	5	0.9947	0.61	1.000
3VPD	24	1-A/B	5	0.9943	0.66	1.000
3VPD	24	2-A/B	3	0.9946	0.64	1.000

PDB ID:	Recycle Number:	Model Number and PDB Reference Chain:	Rank:	TMscore:	RMSD (Å):	Sequence Identity:
3VPD	24	3-A/B	2	0.9946	0.64	1.000
3VPD	24	4-A/B	1	0.9945	0.64	1.000
3VPD	24	5-A/B	4	0.9945	0.64	1.000
5IG9	24	1-A/B	4	0.9782	3.33	0.981
5IG9	24	2-A/B	2	0.9781	3.35	0.981
5IG9	24	3-A/B	5	0.9781	3.35	0.981
5IG9	24	4-A/B	3	0.9783	3.40	0.981
5IG9	24	5-A/B	1	0.9779	3.36	0.981
5IG9	24	1-C/D	4	0.9838	2.36	0.984
5IG9	24	2-C/D	2	0.9836	2.38	0.984
5IG9	24	3-C/D	5	0.9837	2.38	0.984
5IG9	24	4-C/D	3	0.9839	2.42	0.984
5IG9	24	5-C/D	1	0.9835	2.38	0.984
5IG9	24	1-E/H	4	0.9813	2.62	0.982
5IG9	24	2-E/H	2	0.9812	2.64	0.982
5IG9	24	3-E/H	5	0.9812	2.64	0.982
5IG9	24	4-E/H	3	0.9814	2.69	0.982
5IG9	24	5-E/H	1	0.9810	2.65	0.982
5IG9	24	1-F/G	4	0.9829	2.66	0.984
5IG9	24	2-F/G	2	0.9827	2.69	0.984
5IG9	24	3-F/G	5	0.9828	2.69	0.984
5IG9	24	4-F/G	3	0.9829	2.74	0.984
5IG9	24	5-F/G	1	0.9827	2.70	0.984
7DRM	24	1-A/B	4	0.9756	1.45	1.000
7DRM	24	2-A/B	3	0.9752	1.44	1.000
7DRM	24	3-A/B	1	0.9743	1.50	1.000
7DRM	24	4-A/B	5	0.9776	1.37	1.000
7DRM	24	5-A/B	2	0.9746	1.49	1.000
7DRM	24	1-C/D	4	0.9694	1.68	0.997
7DRM	24	2-C/D	3	0.9669	1.86	0.997
7DRM	24	3-C/D	1	0.9669	1.86	0.997
7DRM	24	4-C/D	5	0.9690	1.78	0.997
7DRM	24	5-C/D	2	0.9687	1.71	0.997
7M4S	24	1-A/D	1	0.9445	3.44	1.000
7M4S	24	2-A/D	5	0.9438	3.33	1.000
7M4S	24	3-A/D	4	0.9438	3.33	1.000
7M4S	24	4-A/D	2	0.9465	3.47	1.000
7M4S	24	5-A/D	3	0.9450	3.34	1.000
7M4S	24	1-B/C	1	0.9399	3.24	1.000
7M4S	24	2-B/C	5	0.9411	3.07	1.000
7M4S	24	3-B/C	4	0.9455	3.00	1.000
7M4S	24	4-B/C	2	0.9428	3.24	1.000
7M4S	24	5-B/C	3	0.9411	3.07	1.000
7MGV	24	1-A/B	5	0.9609	2.95	1.000
7MGV	24	2-A/B	1	0.9591	3.05	1.000
7MGV	24	3-A/B	4	0.9580	3.03	1.000
7MGV	24	4-A/B	3	0.9591	3.09	1.000
7MGV	24	5-A/B	2	0.9601	3.03	1.000
3VPB	48	1-A/B	5	0.9920	0.75	1.000
3VPB	48	2-A/B	3	0.9919	0.76	1.000
3VPB	48	3-A/B	1	0.9917	0.77	1.000
3VPB	48	4-A/B	2	0.9925	0.73	1.000
3VPB	48	5-A/B	4	0.9926	0.73	1.000
3VPB	48	1-C/D	5	0.9942	0.64	1.000
3VPB	48	2-C/D	3	0.9957	0.55	1.000
3VPB	48	3-C/D	1	0.9936	0.67	1.000
3VPB	48	4-C/D	2	0.9952	0.58	1.000
3VPB	48	5-C/D	4	0.9947	0.61	1.000
3VPD	48	1-A/B	5	0.9944	0.65	1.000
3VPD	48	2-A/B	4	0.9946	0.64	1.000
3VPD	48	3-A/B	3	0.9946	0.64	1.000
3VPD	48	4-A/B	1	0.9945	0.64	1.000
3VPD	48	5-A/B	2	0.9945	0.64	1.000
5IG9	48	1-A/B	3	0.9782	3.33	0.981

PDB ID:	Recycle Number:	Model Number and PDB Reference Chain:	Rank:	TMscore:	RMSD (Å):	Sequence Identity:
5IG9	48	2-A/B	1	0.9781	3.36	0.981
5IG9	48	3-A/B	5	0.9780	3.36	0.981
5IG9	48	4-A/B	4	0.9781	3.37	0.981
5IG9	48	5-A/B	2	0.9779	3.37	0.981
5IG9	48	1-C/D	3	0.9838	2.36	0.984
5IG9	48	2-C/D	1	0.9836	2.38	0.984
5IG9	48	3-C/D	5	0.9836	2.38	0.984
5IG9	48	4-C/D	4	0.9836	2.40	0.984
5IG9	48	5-C/D	2	0.9835	2.39	0.984
5IG9	48	1-E/H	3	0.9813	2.62	0.982
5IG9	48	2-E/H	1	0.9812	2.65	0.982
5IG9	48	3-E/H	5	0.9812	2.65	0.982
5IG9	48	4-E/H	4	0.9812	2.66	0.982
5IG9	48	5-E/H	2	0.9810	2.66	0.982
5IG9	48	1-F/G	3	0.9829	2.67	0.984
5IG9	48	2-F/G	1	0.9827	2.70	0.984
5IG9	48	3-F/G	5	0.9828	2.70	0.984
5IG9	48	4-F/G	4	0.9827	2.71	0.984
5IG9	48	5-F/G	2	0.9827	2.71	0.984
7DRM	48	1-A/B	4	0.9752	1.46	1.000
7DRM	48	2-A/B	3	0.9741	1.48	1.000
7DRM	48	3-A/B	1	0.9749	1.47	1.000
7DRM	48	4-A/B	5	0.9775	1.37	1.000
7DRM	48	5-A/B	2	0.9747	1.48	1.000
7DRM	48	1-C/D	4	0.9689	1.71	0.997
7DRM	48	2-C/D	3	0.9658	1.90	0.997
7DRM	48	3-C/D	1	0.9664	1.81	0.997
7DRM	48	4-C/D	5	0.9691	1.78	0.997
7DRM	48	5-C/D	2	0.9686	1.72	0.997
7M4S	48	1-A/D	1	0.9435	3.44	1.000
7M4S	48	2-A/D	3	0.9429	3.34	1.000
7M4S	48	3-A/D	5	0.9486	3.23	1.000
7M4S	48	4-A/D	2	0.9460	3.46	1.000
7M4S	48	5-A/D	4	0.9439	3.35	1.000
7M4S	48	1-B/C	1	0.9393	3.23	1.000
7M4S	48	2-B/C	3	0.9413	3.04	1.000
7M4S	48	3-B/C	5	0.9449	2.99	1.000
7M4S	48	4-B/C	2	0.9420	3.25	1.000
7M4S	48	5-B/C	4	0.9403	3.08	1.000
7MGV	48	1-A/B	3	0.9596	2.98	1.000
7MGV	48	2-A/B	1	0.9580	3.07	1.000
7MGV	48	3-A/B	5	0.9565	3.04	1.000
7MGV	48	4-A/B	4	0.9577	3.12	1.000
7MGV	48	5-A/B	2	0.9571	3.07	1.000
3VPB	72	1-A/B	5	0.9920	0.76	1.000
3VPB	72	2-A/B	3	0.9918	0.76	1.000
3VPB	72	3-A/B	2	0.9915	0.78	1.000
3VPB	72	4-A/B	1	0.9926	0.73	1.000
3VPB	72	5-A/B	4	0.9924	0.74	1.000
3VPB	72	1-C/D	5	0.9942	0.64	1.000
3VPB	72	2-C/D	3	0.9957	0.55	1.000
3VPB	72	3-C/D	2	0.9936	0.67	1.000
3VPB	72	4-C/D	1	0.9953	0.58	1.000
3VPB	72	5-C/D	4	0.9946	0.62	1.000
3VPD	72	1-A/B	5	0.9944	0.65	1.000
3VPD	72	2-A/B	4	0.9947	0.63	1.000
3VPD	72	3-A/B	2	0.9947	0.63	1.000
3VPD	72	4-A/B	1	0.9946	0.64	1.000
3VPD	72	5-A/B	3	0.9946	0.64	1.000
5IG9	72	1-A/B	4	0.9782	3.33	0.981
5IG9	72	2-A/B	2	0.9779	3.35	0.981
5IG9	72	3-A/B	5	0.9780	3.36	0.981
5IG9	72	4-A/B	3	0.9780	3.36	0.981
5IG9	72	5-A/B	1	0.9778	3.36	0.981

PDB ID:	Recycle Number:	Model Number and PDB Reference Chain:	Rank:	TMscore:	RMSD (Å):	Sequence Identity:
5IG9	72	1-C/D	4	0.9838	2.36	0.984
5IG9	72	2-C/D	2	0.9835	2.38	0.984
5IG9	72	3-C/D	5	0.9836	2.39	0.984
5IG9	72	4-C/D	3	0.9836	2.39	0.984
5IG9	72	5-C/D	1	0.9834	2.39	0.984
5IG9	72	1-E/H	4	0.9812	2.62	0.982
5IG9	72	2-E/H	2	0.9810	2.64	0.982
5IG9	72	3-E/H	5	0.9811	2.65	0.982
5IG9	72	4-E/H	3	0.9811	2.65	0.982
5IG9	72	5-E/H	1	0.9809	2.65	0.982
5IG9	72	1-F/G	4	0.9829	2.67	0.984
5IG9	72	2-F/G	2	0.9827	2.69	0.984
5IG9	72	3-F/G	5	0.9827	2.70	0.984
5IG9	72	4-F/G	3	0.9827	2.70	0.984
5IG9	72	5-F/G	1	0.9826	2.70	0.984
7DRM	72	1-A/B	4	0.9748	1.47	1.000
7DRM	72	2-A/B	3	0.9749	1.45	1.000
7DRM	72	3-A/B	1	0.9750	1.47	1.000
7DRM	72	4-A/B	5	0.9795	1.30	1.000
7DRM	72	5-A/B	2	0.9747	1.48	1.000
7DRM	72	1-C/D	4	0.9685	1.72	0.997
7DRM	72	2-C/D	3	0.9664	1.89	0.997
7DRM	72	3-C/D	1	0.9665	1.81	0.997
7DRM	72	4-C/D	5	0.9707	1.73	0.997
7DRM	72	5-C/D	2	0.9687	1.71	0.997
7M4S	72	1-A/D	2	0.9439	3.44	1.000
7M4S	72	2-A/D	4	0.9436	3.34	1.000
7M4S	72	3-A/D	5	0.9493	3.23	1.000
7M4S	72	4-A/D	1	0.9467	3.47	1.000
7M4S	72	5-A/D	3	0.9477	3.26	1.000
7M4S	72	1-B/C	2	0.9394	3.24	1.000
7M4S	72	2-B/C	4	0.9415	3.04	1.000
7M4S	72	3-B/C	5	0.9454	3.00	1.000
7M4S	72	4-B/C	1	0.9425	3.26	1.000
7M4S	72	5-B/C	3	0.9429	3.03	1.000
7MGV	72	1-A/B	5	0.9607	2.96	1.000
7MGV	72	2-A/B	1	0.9589	3.05	1.000
7MGV	72	3-A/B	3	0.9580	3.02	1.000
7MGV	72	4-A/B	4	0.9582	3.11	1.000
7MGV	72	5-A/B	2	0.9581	3.06	1.000

Table S7. All Non-ATP Grasp Ligase RiPP biosynthetic enzyme US-align results. Enzymes with multiple available reference subunits are notated with their corresponding chain letters.

PDB ID:	Model Number:	Rank:	TMscore:	RMSD (Å):	Sequence Identity:
**5DZT**	1	4	0.99536	0.72	1.000
**5DZT**	2	1	0.99491	0.78	1.000
**5DZT**	3	3	0.92851	2.92	1.000
**5DZT**	4	5	0.92207	3.09	1.000
**5DZT**	5	2	0.92397	3.06	1.000
**4WD9**	1	5	0.99284	3.94	0.994
**4WD9**	2	4	0.99228	3.94	0.994
**4WD9**	3	1	0.99201	3.96	0.994
**4WD9**	4	3	0.99226	3.91	0.994
**4WD9**	5	2	0.99268	3.94	0.994
**5W99**	1	1	0.99725	0.34	1.000
**5W99**	2	2	0.99652	0.39	1.000
**5W99**	3	4	0.98235	0.93	1.000
**5W99**	4	5	0.97966	1.02	1.000
**5W99**	5	3	0.98630	0.83	1.000
**6EC8**	1	2	0.99462	0.81	1.000

PDB ID:	Model Number:	Rank:	TMscore:	RMSD (Å):	Sequence Identity:
**6EC8**	2	1	0.99440	0.84	1.000
**6EC8**	3	3	0.96277	1.99	1.000
**6EC8**	4	5	0.9570	2.15	1.000
**6EC8**	5	4	0.9659	1.92	1.000
**4BS9**	1	4	0.9571	2.03	1.000
**4BS9**	2	2	0.9596	1.97	1.000
**4BS9**	3	3	0.9526	2.14	1.000
**4BS9**	4	5	0.9505	2.18	1.000
**4BS9**	5	1	0.9448	2.31	1.000
**6PEU**	1-A/B	2	0.9760	1.50	1.000
**6PEU**	2-A/B	1	0.9771	1.46	1.000
**6PEU**	3-A/B	3	0.9331	2.59	1.000
**6PEU**	4-A/B	5	0.8184	4.70	1.000
**6PEU**	5-A/B	4	0.8728	3.76	1.000
**6PEU**	1-C/D	2	0.9746	2.64	1.000
**6PEU**	2-C/D	1	0.9763	2.61	1.000
**6PEU**	3-C/D	3	0.9369	3.27	1.000
**6PEU**	4-C/D	5	0.8062	5.26	1.000
**6PEU**	5-C/D	4	0.8642	4.38	1.000
**4H6V**	1	5	0.9587	2.58	1.000
**4H6V**	2	2	0.9589	2.59	1.000
**4H6V**	3	1	0.9549	2.63	1.000
**4H6V**	4	4	0.9532	2.70	1.000
**4H6V**	5	3	0.9537	2.69	1.000
**5N0Q**	1	3	0.9875	1.91	0.996
**5N0Q**	2	5	0.9892	1.86	0.996
**5N0Q**	3	2	0.9890	1.87	0.996
**5N0Q**	4	4	0.9897	1.85	0.996
**5N0Q**	5	1	0.9896	1.85	0.996
**5FF5**	1	3	0.9736	1.81	1.000
**5FF5**	2	1	0.9757	1.74	1.000
**5FF5**	3	4	0.9592	2.24	1.000
**5FF5**	4	5	0.9719	1.85	1.000
**5FF5**	5	2	0.9753	1.75	1.000
**6JX3**	1	3	0.9856	0.40	1.000
**6JX3**	2	4	0.9818	0.46	1.000
**6JX3**	3	2	0.9589	0.78	1.000
**6JX3**	4	1	0.9577	0.81	1.000
**6JX3**	5	5	0.9598	0.79	1.000
**6OM4**	1-A	2	0.9849	0.85	1.000
**6OM4**	2-A	1	0.9856	0.82	1.000
**6OM4**	3-A	4	0.9685	1.35	1.000
**6OM4**	4-A	5	0.9611	1.72	1.000
**6OM4**	5-A	3	0.9720	1.23	1.000
**6OM4**	1-B	2	0.9899	0.69	1.000
**6OM4**	2-B	1	0.9900	0.68	1.000
**6OM4**	3-B	4	0.9672	1.42	1.000
**6OM4**	4-B	5	0.9578	1.84	1.000
**6OM4**	5-B	3	0.9704	1.26	1.000
**4KVZ**	1	1	0.9754	1.29	1.000
**4KVZ**	2	2	0.9765	1.23	1.000
**4KVZ**	3	3	0.9775	1.14	1.000
**4KVZ**	4	5	0.9760	1.20	1.000
**4KVZ**	5	4	0.9772	1.14	1.000
**4KWC**	1	3	0.9953	0.41	1.000
**4KWC**	2	1	0.9953	0.41	1.000
**4KWC**	3	4	0.9896	0.62	1.000
**4KWC**	4	5	0.9900	0.61	1.000
**4KWC**	5	2	0.9844	0.77	1.000
**6JDD**	1	3	0.9980	0.23	1.000
**6JDD**	2	5	0.9978	0.24	1.000
**6JDD**	3	2	0.9710	1.32	1.000
**6JDD**	4	4	0.9734	1.31	1.000
**6JDD**	5	1	0.9724	1.30	1.000
**5TTY**	1	2	0.9847	1.05	1.000

PDB ID:	Model Number:	Rank:	TMscore:	RMSD (Å):	Sequence Identity:
**5TTY**	2	1	0.9851	1.02	1.000
**5TTY**	3	4	0.9741	1.22	1.000
**5TTY**	4	5	0.9697	1.33	1.000
**5TTY**	5	3	0.9754	1.17	1.000
**6C0Y**	1-A/E	3	0.9949	0.40	1.000
**6C0Y**	2-A/E	1	0.9951	0.39	1.000
**6C0Y**	3-A/E	4	0.9949	0.40	1.000
**6C0Y**	4-A/E	5	0.9949	0.40	1.000
**6C0Y**	5-A/E	2	0.9952	0.38	1.000
**6C0Y**	1-C/G	3	0.9926	0.48	1.000
**6C0Y**	2-C/G	1	0.9917	0.51	1.000
**6C0Y**	3-C/G	4	0.9910	0.53	1.000
**6C0Y**	4-C/G	5	0.9923	0.49	1.000
**6C0Y**	5-C/G	2	0.9918	0.50	1.000
**6C0Y**	1-B/F	3	0.9949	0.40	1.000
**6C0Y**	2-B/F	1	0.9952	0.38	1.000
**6C0Y**	3-B/F	4	0.9950	0.39	1.000
**6C0Y**	4-B/F	5	0.9949	0.40	1.000
**6C0Y**	5-B/F	2	0.9953	0.38	1.000
**6C0Y**	1-H/D	3	0.9937	0.44	1.000
**6C0Y**	2-H/D	1	0.9938	0.44	1.000
**6C0Y**	3-H/D	4	0.9932	0.46	1.000
**6C0Y**	4-H/D	5	0.9937	0.44	1.000
**6C0Y**	5-H/D	2	0.9938	0.44	1.000

Figure S3. Comparison of change in TMscore and RMSD upon changing parameters.

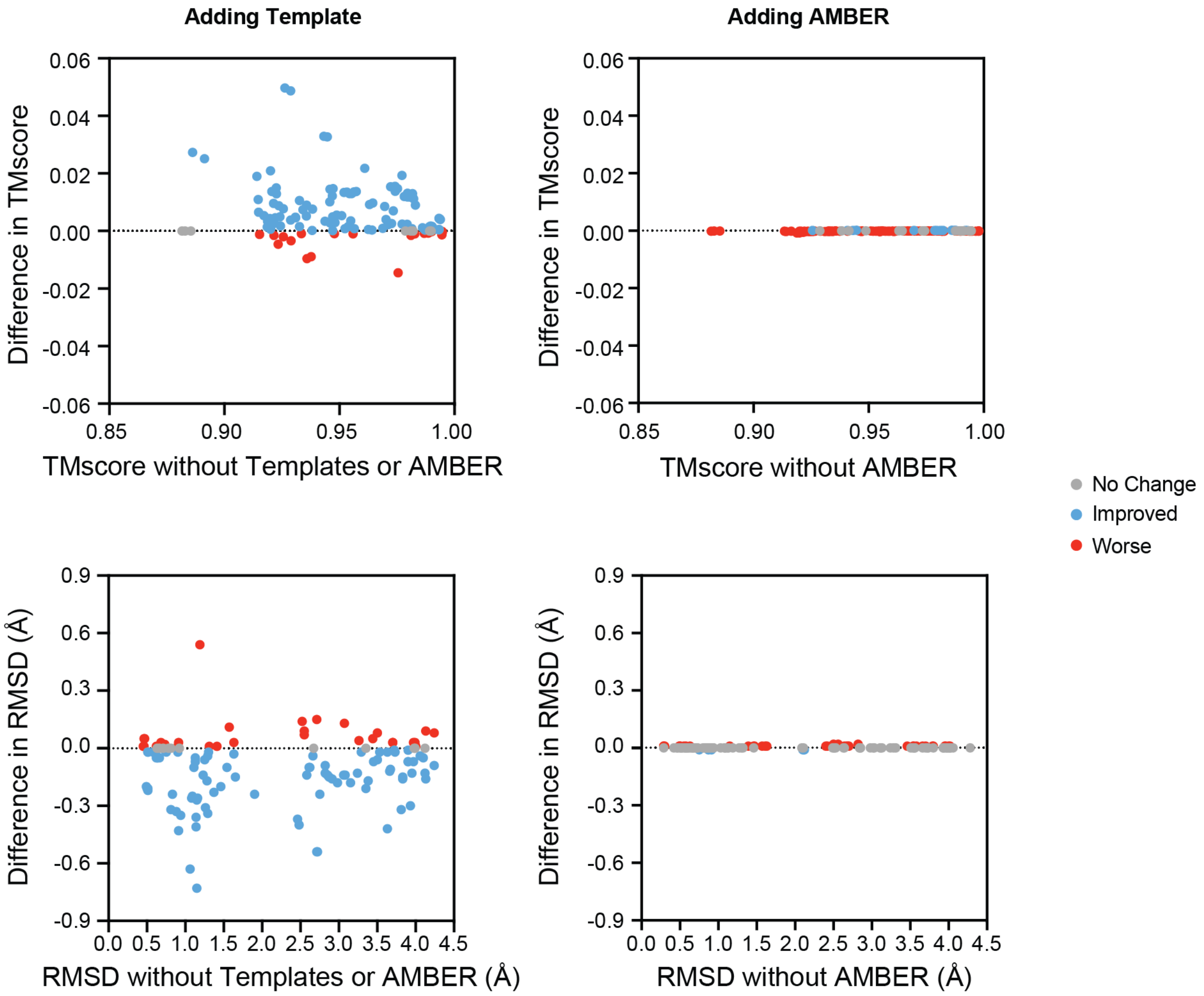

Figure S4. Comparison of monomer structure predictions to each other. Mean TMscores and RMSD are represented by the horizontal lines. Error bars represent standard deviations, and gray circles are values from individual comparisons.

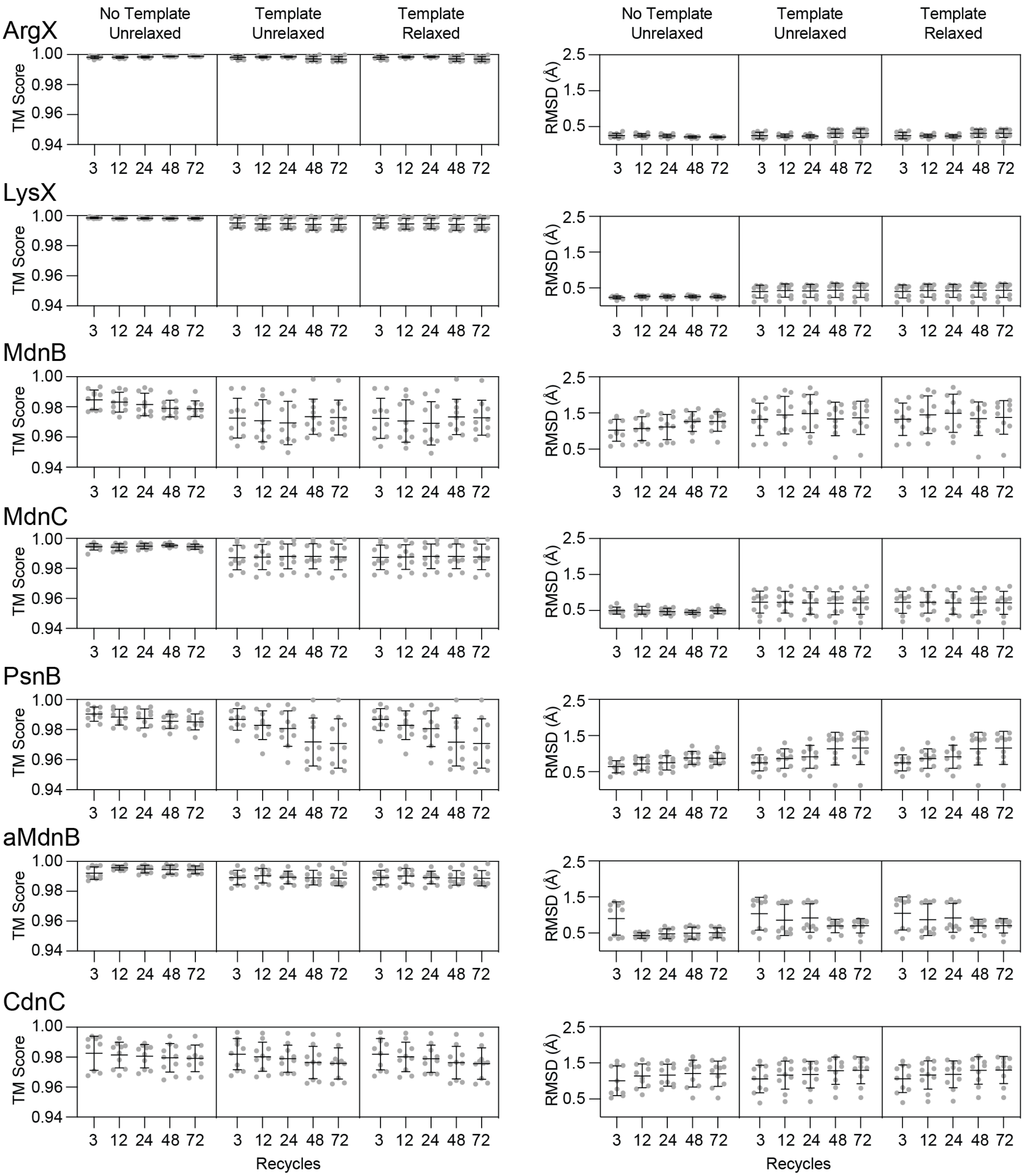

Figure S5. All Non-ATP Grasp Ligase RiPP biosynthetic enzyme predicted models aligned to reported PDB structure (yellow)

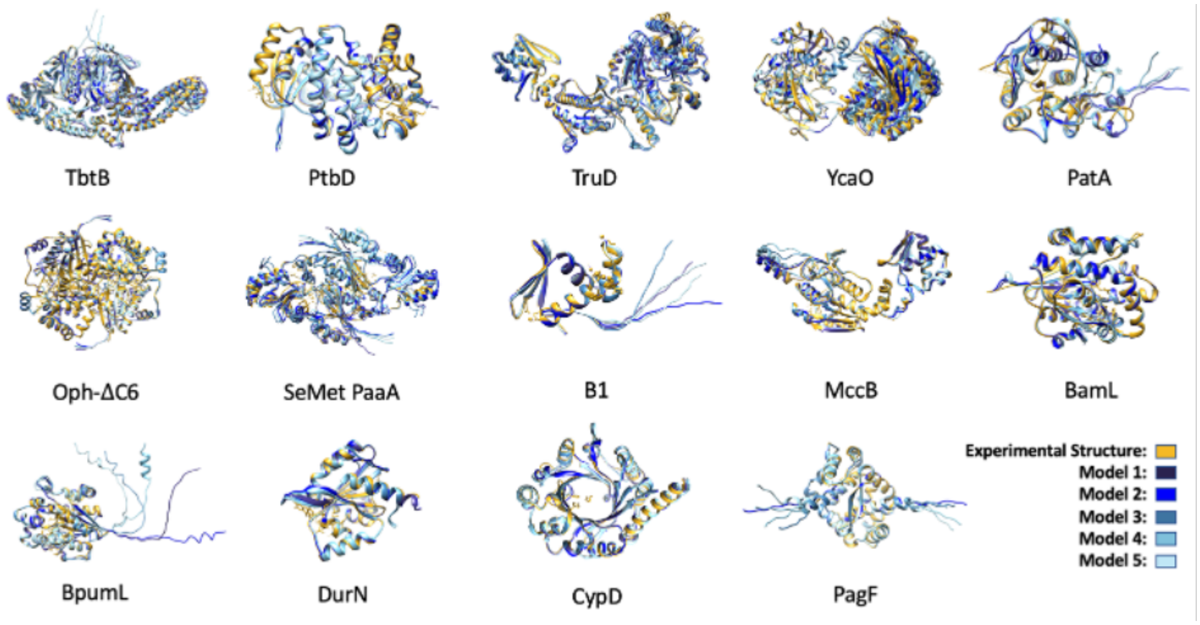

Supplement: Supplementary file 1 [file biomolecules-13-01243-s001.zip › biomolecules-2545106-supplementary.pdf]
